# Metabolic Responses to Cyclic Fasting in Juvenile Turbot (*Scophthalmus maximus*)

**DOI:** 10.1155/anu/7599690

**Published:** 2026-06-25

**Authors:** Haiyan Xiong, Zhengwei Ye, Chenchen Bian, Yanjiao Zhang, Jiahao Liu, Qiang Ma, Yuliang Wei, Mengqing Liang, Houguo Xu

**Affiliations:** ^1^ Key Laboratory of Aquaculture Nutrition and Feed (Ministry of Agriculture) and Key Laboratory of Mariculture (Ministry of Education), Ocean University of China, Qingdao, 266003, China, ouc.edu.cn; ^2^ State Key Laboratory of Mariculture Biobreeding and Sustainable Goods, Yellow Sea Fisheries Research Institute, Chinese Academy of Fishery Sciences, Qingdao, 266071, China, cafs.ac.cn

**Keywords:** compensatory growth, lipid composition, metabolic adaptation, nutritional regulation, starvation

## Abstract

Feed accounts for the majority of aquaculture production costs, making strategies that reduce feed input without compromising growth economically attractive. This 72‐day study evaluated the effects of cyclic fasting regimens on growth, body composition, and liver transcriptome in juvenile turbot (initial body weight 5.54 g). Fish were assigned to a control group (CON) (continuously fed) and three cyclic fasting groups: fasting for 1 day followed by refeeding for 3 days (FT1RF3), fasting for 2 days followed by refeeding for 6 days (FT2RF6), and fasting for 3 days followed by refeeding for 9 days (FT3RF9). The FT1RF3 regimen showed potential for inducing compensatory growth, with final body weight comparable to that of the CON and a numerically lower feed conversion ratio. Minimal differences in protein, amino acid, and fatty acid compositions were observed across groups. FT1RF3 was associated with broad metabolic suppression, enhanced fatty acid utilization, heightened innate immune preparedness, and promotion of cellular renewal. In contrast, FT2RF6 and FT3RF9 resulted in systemic lipid accumulation and numerically lower final body weights, with the metabolic strategy appearing to shift toward survival‐oriented energy storage at the expense of somatic growth. Overall, juvenile turbot exhibited period‐dependent responses to fasting: FT1RF3 showed potential for inducing compensatory growth through metabolic adaptation, whereas fasting for 2–3 days suggested a tendency toward metabolic dysfunction and growth suppression. These findings provide a scientific basis for applying cyclic fasting regimens in aquaculture, with fasting durations tailored to fish developmental stages.

## 1. Introduction

Achieving optimal growth and feed conversion efficiency is fundamental to the profitability in commercial aquaculture. Improving feed efficiency while maintaining growth performance has therefore become a central objective of aquaculture nutrition research, prompting considerable interest in cost‐saving feeding strategies. Compensatory growth, the accelerated growth observed upon refeeding following a period of feed deprivation, offers a promising approach to reduce feed costs without compromising production outcomes [1–4]. For instance, cyclic feed restriction over 2 months in red‐claw crayfish (*Cherax quadricarinatus*) and pacu (*Piaractus mesopotamicus*) achieved roughly 50% feed savings with no loss of biomass production [5, 6]. In Pacific white shrimp (*Penaeus vannamei*) reared in biofloc systems, similar feeding strategies reduced feed costs by nearly 25% [7]. Compensatory growth has also been linked to improved flesh quality in terms of nutritional value and texture, further enhancing its appeal to the aquaculture industry [6]. Therefore, seeking compensatory growth through fasting strategies has been an important measure to improve the production efficiency of aquaculture activities. From a practical production perspective, feed deprivation strategies can be categorized into two types: single continuous fasting and cyclic fasting, each with distinct applications. Single continuous fasting, defined as a prolonged fasting period followed by refeeding, is primarily applicable to specific emergency situations or pretreatments, such as overwintering fasting, intestinal cleansing prior to long‐distance transport, reduced feed intake during disease outbreaks, and flesh quality purification before harvest [8]. In these scenarios, maximizing growth is not the primary objective, and compensatory growth may not occur or may even be temporarily sacrificed. Cyclic fasting, which involves multiple fasting‐refeeding cycles throughout the production cycle, demonstrates greater practical value in routine production management. This strategy can be integrated into daily production rhythms, reactivating compensatory responses in each cycle and thereby extending the restriction period far beyond what single continuous fasting can achieve [9]. Studies have shown that appropriately designed cyclic fasting can induce complete compensatory growth in multiple fish species. For example, rainbow trout (*Oncorhynchus mykiss*) subjected to 1–2 days of fasting per week for 10 weeks achieved final body weights comparable to continuously fed controls [10]. Similarly, Nile tilapia (*Oreochromis niloticus*) reared in biofloc systems under cyclic regimens of 6:12 and 12:36 days (fasting:refeeding) for 20 weeks also exhibited full compensatory growth [11]. In the sailfin molly (*Poecilia latipinna*), cycles of 3 days of fasting followed by 6 days of refeeding over 54 days triggered compensatory growth [12]. In contrast, longfin yellowtail (*Seriola rivoliana*) failed to achieve compensatory growth under cycles of 3 days of fasting and 7 days of refeeding over 85 days, whereas a 2:1 fasting:refeeding cycle applied over 89 days resulted in partial compensatory growth [13, 14]. Collectively, these findings indicate that the occurrence of compensatory growth under similar fasting:refeeding ratios is highly species‐specific. Differences in compensatory growth are often closely related to species‐specific metabolic characteristics in particular energy reserve, feeding habits, and culture environments. The life history of turbot differs from that of other known fish species, and thus, findings from those species cannot be directly applied. Given its economic value, further investigation on turbot is warranted.

In most previous studies on cyclic fasting, divergent fasting–refeeding ratios across treatment groups inevitably cause discrepancies in total fasting days. This made it difficult to distinguish the effects of individual fasting duration from those of cumulative feed deprivation. This study examined three cyclic fasting regimens in juvenile turbot within a 72‐day trial: fasting for 1 day followed by refeeding for 3 days (FT1RF3), fasting for 2 days followed by refeeding for 6 days (FT2RF6), and fasting for 3 days followed by refeeding for 9 days (FT3RF9). All treatments shared a consistent 1:3 fasting–refeeding ratio, which was screened by a preliminary experiment. All these treatments yielded 18 fasting days and 54 refeeding days in total. The only variable across treatments was the duration of each individual fasting period. Growth performance, body composition, and liver transcriptomes were analyzed to identify optimal fasting regimens and clarify relevant metabolic responses.

## 2. Materials and Methods

### 2.1. Experimental Design and Sampling

**Figure 1:**
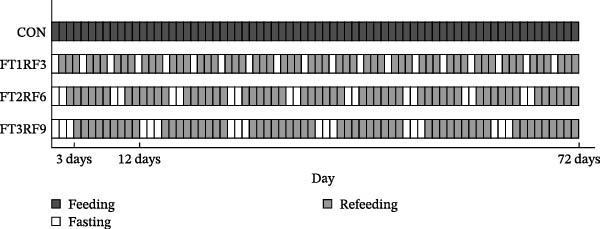
Schematic chart of the four feeding regimes for experimental turbot. CON, continuous feeding without fasting; FT1RF3, fasting for 1 day followed by refeeding for 3 days; FT2RF6, fasting for 2 days followed by refeeding for 6 days; FT3RF9, fasting for 3 days followed by refeeding for 9 days (*n* = 3).

Juvenile turbots (initial body weight: 5.54 g) were obtained from Haiyang Huanghai Aquaculture Co., Ltd. (Yantai, China) and acclimated for 7 days in experimental tanks (57.0 cm× 39.0 cm× 35.0 cm). A total of 240 fish were randomly distributed into 12 tanks (20 fish per tank) and assigned to four experimental groups with three replicate tanks per group. For the 72‐day feeding trial, fish were fed a commercial diet (Table 1) (proximate composition: moisture, 12.00%; crude protein, 54.00%; crude lipid, 8.00%; ash, 14.96%; and MJ/kg gross energy, 21.60; manufactured by Qingdao Surgreen Bioengineering Co., Ltd., Qingdao, China) under distinct feeding regimes: a continuously fed control group (CON) and three cyclic fasting–refeeding groups: FT1RF3, FT2RF6, and FT3RF9 (Figure 1). Throughout the trial, when the fish were fed, they were hand‐fed to apparent satiation (feed pellets sank to the bottom and were no longer actively ingested) twice daily (08:00 and 20:00). Water quality parameters were maintained within the following ranges: water temperature, 16–20°C; salinity, 29–30; dissolved oxygen, >7 mg/L; and pH, 7.5–8.5.

**Table 1:** Amino acid and fatty acid compositions of the experimental diets.

Amino acid	Concentration	Fatty acid	Concentration
Dry matter basis (%)		Total fatty acids (%)	
Threonine	2.42	Myristic acid (14:0)	4.72
Valine	2.64	Palmitic acid (16:0)	21.70
Methionine	1.37	Stearic acid (18:0)	4.52
Isoleucine	2.42	SFA	31.00
Leucine	4.19	Palmitoleic acid (16:1n‐7)	3.90
Phenylalanine	2.13	Oleic acid (18:1n‐9)	14.95
Lysine	4.34	Eicosenoic acid (20:1n‐9)	1.84
Histidine	1.69	MUFA	20.69
Arginine	3.08	Linoleic acid (18:2n‐6)	13.03
TEAA	24.28	Eicosadienoic acid (20:2n‐6)	0.26
Aspartic acid	5.10	Arachidonic acid (20:4n‐6)	0.73
Serine	2.27	n‐6PUFA	14.03
Glutamic acid	8.06	α‐Linolenic acid (18:3n‐3)	1.85
Glycine	3.41	Eicosapentaenoic acid (20:5n‐3)	7.11
Alanine	3.46	Docosahexaenoic acid (22:6n‐3)	11.12
Cysteine	0.21	n‐3PUFA	20.07
Tyrosine	1.78	—	—
Proline	2.59	—	—
TNEAA	26.88	—	—

Abbreviations: EAAs, essential amino acids; MUFAs, monounsaturated fatty acids; NEAAs, nonessential amino acids; PUFAs, polyunsaturated fatty acids; SFA, saturated fatty acid; TEAAs, total essential amino acids; TNEAAs, total nonessential amino acids.

At the end of the feeding trial, fish were fasted for 12 h after their respective final refeeding phase and then sampled. Subsequent procedures, including anesthesia, tissue dissection (liver, muscle, and subcutaneous adipose tissue near the fin [STF]), blood collection from the caudal vein, and sample cryopreservation, were carried out as described by Xiong et al. [15].

### 2.2. Analytical Methods

#### 2.2.1. Nutrient Composition Analysis

Proximate composition was analyzed following AOAC [16]: moisture (method 934.01), crude protein (method 954.01, using a KJELTEC 2300 analyzer), ash (method 942.05), and whole‐body crude lipid (method 2003.05, using a Soxtec 2050 system) were determined accordingly. Tissue lipid content was analyzed according to Folch et al. [17]. Dietary proximate composition was analyzed by Qingdao Surgreen Bioengineering Co., Ltd. The gross energy was determined using a Parr 6100 oxygen bomb calorimeter (ISO 9831–1998). Fatty acid and amino acid compositions were analyzed using a GC‐2010 Pro (Shimadzu) and a Hitachi L‐8900 amino acid analyzer, respectively [15].

#### 2.2.2. Biochemical Analysis of Serum and Tissues

Serum and tissues were assayed for triglyceride (TG), total cholesterol (T‐CHO), non‐esterified fatty acid (NEFA), glucose, pyruvate, lactate (LD), glycogen, total protein (TP), and total amino acids (TAAs) using commercial kits (Nanjing Jiancheng, China) with an Infinite M200 microplate reader (Tecan, Switzerland), following the manufacturer’s protocols.

#### 2.2.3. Transcriptomic Analysis

Total RNA was extracted from pooled liver samples (10 fish randomly selected per tank) using the TRIzol reagent (TRI‐Reagent, Ambion) according to the manufacturer’s instructions. RNA integrity and concentration were assessed using an Agilent 5400 system. All samples had RIN values ≥ 9 and total RNA > 20 μg. Twelve sequencing libraries (three biological replicates per treatment group) were prepared and, following quality control, sequenced on the Illumina NovaSeq X Plus platform (Novogene Co., Beijing, China) to generate 2 × 150 bp paired‐end reads.

The raw image data were converted into sequence data (reads) by CASAVA base calling. To ensure data quality and reliability, raw reads were processed using fastp (version 0.19.7) to remove adapter‐contaminated reads, low‐quality reads, and reads containing poly‐N. The GC content, Q20, and Q30 values of the clean data were subsequently calculated. All downstream analyses were performed using high‐quality clean data. Clean reads from each of the 12 libraries were mapped to the turbot reference genome (ASM1334776v1) using HISAT2 software (version 2.0.5), and novel transcripts were assembled with StringTie [17]. Transcript abundance for each treatment was quantified per library as fragments per kilobase per million (FPKM) using featureCounts (version 1.5.0‐p3). Differential expression analysis was conducted using the DESeq2 package [18], and genes with |log_2_ (fold change) | > 0 and an adjusted *p*‐value (*p*_adj_) < 0.05 were considered significantly differentially expressed. Kyoto Encyclopedia of Genes and Genomes (KEGG) pathway enrichment analysis of differentially expressed genes (DEGs) was performed using the ClusterProfiler package (version 3.8.1). The sequencing data have been deposited in the NCBI GenBank database under the BioProject accession number PRJNA1434104.

### 2.3. Calculations and Statistical Methods

The growth‐related parameters were calculated as follows:

Weight gain WG,% = Final body weight −initial body weight /initial body weight × 100,

Specific growth rate (SGR, %/d)=lnfinal mean body weight, g−lninitial mean body weight, g/number of days×100,

Feed intake g/fish = Amount of feed consumed g/number of fish,

Survival % =Final fish number/initial fish number × 100,

Feed conversion ratio FCR =Feed intake/weight gain,

Viscerosomatic index VSI % = Wet viscera weight/fish body weight × 100,

Hepatosomatic index HSI % = Wet liver weight/fish body weight × 100,

Condition factor K =Body weight/body length3 × 100.

Statistical analyses were performed using SPSS 25.0 for Windows. Prior to statistical evaluation, data were tested for normality and homogeneity of variance using the Shapiro–Wilk test and Levene’s test, respectively. Differences among groups were examined by one‐way ANOVA, followed by Tukey’s HSD post hoc test. When assumptions of normality or homogeneity of variance were violated, data were log‐transformed or analyzed using the non‐parametric Kruskal–Wallis test. Significance was set at *p*  < 0.05.

## 3. Results

### 3.1. Growth Performance

All fish survived the 72‐day experimental period (Table 2). Fish in the FT1RF3 group achieved final body weight comparable to the CON (*p* > 0.05). The FT2RF6 and FT3RF9 groups showed numerically lower final body weights, although the differences were not statistically significant (*p* > 0.05). The WG and SGR did not differ significantly among groups (*p* > 0.05), though numerical decreases were observed in all cyclic fasting groups compared to CON. Feed intake was significantly lower in the FT2RF6 and FT3RF9 groups compared to CON (*p* < 0.05), but did not differ significantly in the FT1RF3 group (*p* > 0.05), though a numerical decrease was observed (Table 2). Although no statistically significant differences were observed in the FCR among treatments, all cyclic fasting regimens resulted in numerically lower FCR values compared to the CON (Table 2).

**Table 2:** Growth performance and somatic parameters (mean ± standard error).

Parameters	CON	FT1RF3	FT2RF6	FT3RF9
Initial body weight (g)	5.46 ± 0.12	5.54 ± 0.18	5.45 ± 0.04	5.44 ± 0.31
Final body weight (g)	44.07 ± 0.72	43.02 ± 1.79	39.29 ± 0.95	39.12 ± 1.75
Weight gain (%)	708.50 ± 28.1	680.47 ± 54.0	620.90 ± 16.6	623.27 ± 42.1
SGR (%/d)	2.90 ± 0.05	2.85 ± 0.09	2.74 ± 0.03	2.74 ± 0.08
Feed intake (g/fish)	25.50 ± 0.88^b^	21.90 ± 1.16^ab^	20.70 ± 0.59^a^	20.90 ± 1.13^a^
FCR	0.66 ± 0.02	0.58 ± 0.02	0.61 ± 0.03	0.62 ± 0.03
HSI (%)	1.53 ± 0.10	1.50 ± 0.12	1.61 ± 0.06	1.71 ± 0.09
VSI (%)	6.03 ± 0.25	5.36 ± 0.19	6.30 ± 0.28	6.10 ± 0.25
*K* (g (cm)^−3^)	3.19 ± 0.05^a^	3.35 ± 0.08^a^	3.84 ± 0.11^b^	3.44 ± 0.09^a^
Survival (%)	100.00 ± 0.00	100.00 ± 0.00	100.00 ± 0.00	100.00 ± 0.00

*Note:* For HSI, VSI, and *K*, each tank value represents the mean of three individual fish. Within a row, means without a same superscript letter are significantly different (*p* < 0.05).

Abbreviations: CON, continuous feeding; FT1RF3, 1 day fasting followed by 3 days refeeding; FT2RF6, 2 days fasting followed by 6 days refeeding; FT3RF9, 3 days fasting followed by 9 days refeeding (*n* = 3 replicate tanks).

Regarding somatic indices, the HSI and VSI did not differ significantly among groups (*p* > 0.05), though the HSI tended to be higher in the FT2RF6 and FT3RF9 groups and the VSI tended to be lower in the FT1RF3 group. The *K* was significantly higher in the FT2RF6 group compared to all other groups (*p* < 0.05) (Table 2).

### 3.2. Proximate Composition of Fish Body and Tissues

**Figure 2:**
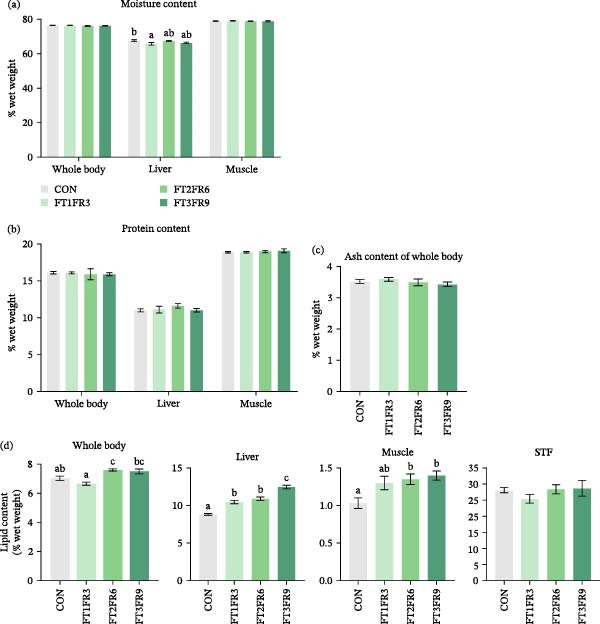
The effects of the experimental feeding regimes on the proximate composition of experimental turbot. (a) Moisture content in whole body, muscle and liver. (b) Protein content of whole body, muscle and liver. (c) Ash content of whole body. (d) lipid content in whole‐body, muscle, liver and subcutaneous adipose tissue near the fin (STF). All data were reported as mean ± standard error. CON, continuous feeding without fasting; FT1RF3, fasting for 1 day followed by refeeding for 3 days; FT2RF6, fasting for 2 days followed by refeeding for 6 days; FT3RF9, fasting for 3 days followed by refeeding for 9 days (*n* = 3 replicate tanks; each tank value is the mean of three fish). For a certain parameter, the data bars without letter or sharing a same letter are not significantly different (*p* > 0.05).

Cyclic fasting had limited effects on moisture and ash content. Liver moisture content was significantly lower in the FT1RF3 group compared to CON (*p* < 0.05), while muscle and whole‐body moisture remained comparable across groups (*p* > 0.05) (Figure 2a). The whole‐body ash content was not affected by cyclic fasting (*p* > 0.05) (Figure 2c). The crude protein content in whole body, muscle, and liver showed no significant differences across all cyclic fasting groups (*p* > 0.05) (Figure 2b).

Whole‐body lipid content was the lowest in the FT1RF3 group, being significantly reduced compared to the FT2RF6 and FT3RF9 groups (*p* < 0.05). The FT2RF6 and FT3RF9 groups exhibited elevated whole‐body lipid content relative to CON, though the differences did not reach statistical significance (*p* > 0.05). The liver and muscle lipid content increased progressively with prolonged single fasting duration, following the rank: FT3RF9 > FT2RF6 > FT1RF3 > CON. Liver crude lipid content was significantly elevated in all cyclic fasting groups compared to CON (*p* < 0.05) (Figure 2d). Muscle crude lipid content was significantly higher in the FT2RF6 and FT3RF9 groups compared to CON (*p* < 0.05). STF lipid content was lower in the FT1RF3 group than in all other groups, while the FT2RF6 and FT3RF9 groups showed levels comparable to CON (*p* > 0.05) (Figure 2d).

### 3.3. Fatty Acid Composition in Fish Tissues

Cyclic fasting regimens had relatively mild effects on tissue fatty acid profiles (Tables 3–5). The liver fatty acid composition remained largely stable across treatments, with only minor variations. Saturated fatty acid (SFA) content was slightly reduced in the FT3RF9 group compared to CON (*p* < 0.05), while monounsaturated fatty acids (MUFAs) and n‐3 polyunsaturated fatty acids (PUFAs) showed no significant differences among groups (Table 3).

**Table 3:** Fatty acid compositions in liver of experimental turbot (% total fatty acids, mean ± standard error).

Fatty acid	CON	FT1RF3	FT2RF6	FT3RF9
Myristic acid (14:0)	4.19 ± 0.12	4.29 ± 0.12	3.99 ± 0.14	3.89 ± 0.09
Palmitic acid (16:0)	15.00 ± 0.16^ab^	14.50 ± 0.26^ab^	15.40 ± 0.22^b^	14.40 ± 0.27^a^
Stearic acid (18:0)	3.06 ± 0.20	2.90 ± 0.15	3.15 ± 0.10	3.04 ± 0.11
SFA	22.20 ± 0.21^b^	21.70 ± 0.22^ab^	22.50 ± 0.25^b^	21.30 ± 0.26^a^
Palmitoleic acid (16:1n–7)	4.31 ± 0.08^ab^	4.42 ± 0.10^b^	4.00 ± 0.07^a^	4.10 ± 0.06^ab^
Oleic acid (18:1n–9)	20.00 ± 0.46	20.50 ± 0.73	20.20 ± 0.51	20.50 ± 0.62
Eicosenoic acid (20:1n–9)	1.73 ± 0.05	1.87 ± 0.08	1.79 ± 0.05	1.87 ± 0.04
MUFA	26.00 ± 0.42	26.80 ± 0.58	26.00 ± 0.45	26.50 ± 0.58
Linoleic acid (18:2n‐6)	19.10 ± 0.61	19.00 ± 0.64	18.80 ± 0.53	19.50 ± 0.63
Eicosadienoic acid (20:2n‐6)	1.82 ± 0.07	1.72 ± 0.08	1.96 ± 0.14	1.75 ± 0.09
Arachidonic acid (20:4n‐6)	0.89 ± 0.04	0.78 ± 0.02	0.85 ± 0.03	0.83 ± 0.03
n‐6PUFA	21.80 ± 0.58	22.30 ± 0.31	22.60 ± 0.63	22.80 ± 0.48
α‐Linolenic acid (18:3n‐3)	1.60 ± 0.06	1.67 ± 0.08	1.79 ± 0.11	1.61 ± 0.07
Eicosapentaenoic acid (20:5n‐3)	3.57 ± 0.05	3.73 ± 0.13	3.52 ± 0.10	3.47 ± 0.26
Docosahexaenoic acid (22:6n‐3)	10.00 ± 0.11	9.67 ± 0.16	10.30 ± 0.36	10.20 ± 0.17
n‐3PUFA	15.20 ± 0.12	15.10 ± 0.21	15.60 ± 0.39	15.20 ± 0.36

*Note:* (*n* = 3 replicate tanks; each tank value is the mean of three fish). Within a row, means without a same superscript letter are significantly different (*p* < 0.05).

Abbreviations: CON, continuous feeding; FT1RF3, 1 day fasting followed by 3 days refeeding; FT2RF6, 2 days fasting followed by 6 days refeeding; FT3RF9, 3 days fasting followed by 9 days refeeding; MUFAs, monounsaturated fatty acids; PUFAs, polyunsaturated fatty acids. SFA, saturated fatty acid.

**Table 4:** Fatty acid compositions in muscle of experimental turbot (% total fatty acids, mean ± standard error).

Fatty acid	CON	FT1RF3	FT2RF6	FT3RF9
Myristic acid (14:0)	2.87 ± 0.16	2.95 ± 0.22	2.57 ± 0.26	2.19 ± 0.14
Palmitic acid (16:0)	20.35 ± 0.22	20.46 ± 0.22	20.87 ± 0.18	20.99 ± 0.14
Stearic acid (18:0)	5.09 ± 0.25	4.87 ± 0.16	5.58 ± 0.26	4.70 ± 0.94
SFA	28.30 ± 0.33	28.27 ± 0.23	29.02 ± 0.21	27.89 ± 1.00
Palmitoleic acid (16:1n‐7)	2.57 ± 0.18^ab^	2.84 ± 0.17^b^	2.20 ± 0.24^ab^	2.02 ± 0.18^a^
Oleic acid (18:1n‐9)	13.55 ± 0.29	13.72 ± 0.36	12.97 ± 0.54	12.67 ± 0.37
Eicosenoic acid (20:1n‐9)	1.19 ± 0.08	1.31 ± 0.08	1.13 ± 0.10	1.04 ± 0.07
MUFA	17.43 ± 0.51^b^	17.84 ± 0.44^b^	16.48 ± 0.46^ab^	15.39 ± 0.40^a^
Linoleic acid (18:2n‐6)	10.51 ± 0.32	10.66 ± 0.31	10.54 ± 0.31	10.16 ± 0.20
Eicosadienoic acid (20:2n‐6)	0.59 ± 0.01	0.54 ± 0.02	0.58 ± 0.02	0.55 ± 0.02
Arachidonic acid (20:4n‐6)	1.12 ± 0.04^ab^	1.08 ± 0.04^a^	1.22 ± 0.06^ab^	1.27 ± 0.04^b^
n‐6PUFA	12.2 ± 0.29	12.3 ± 0.30	12.3 ± 0.26	12.0 ± 0.2
α‐Linolenic acid (18:3n‐3)	1.32 ± 0.04	1.44 ± 0.04	1.35 ± 0.06	1.28 ± 0.04
Eicosapentaenoic acid (20:5n‐3)	7.09 ± 0.09^ab^	7.15 ± 0.10^b^	6.76 ± 0.08^a^	6.98 ± 0.09^ab^
Docosahexaenoic acid (22:6n‐3)	18.89 ± 0.43^a^	19.39 ± 0.26^ab^	19.32 ± 0.47^ab^	21.16 ± 0.61^b^
n‐3PUFA	27.97 ± 0.71	27.43 ± 0.74	28.09 ± 0.96	29.19 ± 0.63

*Note:* (*n* = 3 replicate tanks; each tank value is the mean of three fish). Within a row, means without a same superscript letter are significantly different (*p* < 0.05).

Abbreviations: CON, continuous feeding; FT1RF3, 1 day fasting followed by 3 days refeeding; FT2RF6, 2 days fasting followed by 6 days refeeding; FT3RF9, 3 days fasting followed by 9 days refeeding; MUFAs, monounsaturated fatty acids; PUFAs, polyunsaturated fatty acids; SFA, saturated fatty acid.

**Table 5:** Fatty acid compositions in subcutaneous adipose tissue around the fin of experimental turbot (% total fatty acids, mean ± standard error).

Fatty acid	CON	FT1RF3	FT2RF6	FT3RF9
Myristic acid (14:0)	4.88 ± 0.13	4.67 ± 0.21	5.00 ± 0.00	4.67 ± 0.21
Palmitic acid (16:0)	16.00 ± 0.00	16.17 ± 0.17	16.33 ± 0.21	16.50 ± 0.22
Stearic acid (18:0)	2.53 ± 0.03^a^	2.73 ± 0.04^b^	2.76 ± 0.04^b^	2.74 ± 0.04^b^
SFA	24.01 ± 0.07^a^	24.58 ± 0.14^ab^	24.93 ± 0.18^b^	24.61 ± 0.21^b^
Palmitoleic acid (16:1n‐7)	5.30 ± 0.12	5.36 ± 0.13	5.44 ± 0.16	5.36 ± 0.16
Oleic acid (18:1n‐9)	17.13 ± 0.13	17.12 ± 0.10	17.34 ± 0.16	17.34 ± 0.23
Eicosenoic acid (20:1n‐9)	1.98 ± 0.02	1.93 ± 0.01	2.00 ± 0.03	1.95 ± 0.03
MUFA	24.42 ± 0.15	24.41 ± 0.17	24.78 ± 0.21	24.65 ± 0.31
Linoleic acid (18:2n‐6)	13.59 ± 0.11	13.06 ± 0.15	13.63 ± 0.22	13.46 ± 0.24
Eicosadienoic acid (20:2n‐6)	0.66 ± 0.02^b^	0.58 ± 0.02^a^	0.66 ± 0.02^b^	0.65 ± 0.02^ab^
Arachidonic acid (20:4n‐6)	0.65 ± 0.01	0.65 ± 0.01	0.63 ± 0.02	0.65 ± 0.02
n‐6PUFA	14.90 ± 0.11	14.29 ± 0.16	14.92 ± 0.24	14.76 ± 0.25
α‐Linolenic acid (18:3n‐3)	1.77 ± 0.03^a^	1.82 ± 0.03^ab^	1.92 ± 0.03^b^	1.85 ± 0.05^ab^
Eicosapentaenoic acid (20:5n‐3)	7.32 ± 0.05^b^	7.33 ± 0.11^b^	6.75 ± 0.06^a^	6.88 ± 0.05^a^
Docosahexaenoic acid (22:6n‐3)	11.51 ± 0.11	11.52 ± 0.08	10.99 ± 0.20	11.37 ± 0.30
n‐3PUFA	20.61 ± 0.13^b^	20.67 ± 0.16^b^	19.65 ± 0.19^a^	20.11 ± 0.31^ab^

*Note:* (*n* = 3 replicate tanks; each tank value is the mean of three fish). Within a row, means without a same superscript letter are significantly different (*p* < 0.05).

Abbreviations: CON, continuous feeding; FT1RF3, 1 day fasting followed by 3 days refeeding; FT2RF6, 2 days fasting followed by 6 days refeeding; FT3RF9, 3 days fasting followed by 9 days refeeding, MUFAs, monounsaturated fatty acids; PUFAs, polyunsaturated fatty acids; SFA, saturated fatty acid.

The muscle fatty acid composition was also generally stable across groups. Notably, the DHA (22 : 6n‐3) content was elevated in the FT1RF3 group relative to CON (*p* > 0.05) (Table 4). The MUFA content tended to be lower in the FT2RF6 and FT3RF9 groups, with the FT3RF9 group reaching statistical significance (*p* < 0.05). The EPA (20:5n‐3) content showed a decreasing trend in the FT2RF6 and FT3RF9 groups (Table 4). In STF, the SFA content was significantly higher in the FT2RF6 and FT3RF9 groups compared to CON (*p* < 0.05), primarily driven by increased 18:0 content (Table 5). Conversely, the EPA content was significantly reduced in the FT2RF6 and FT3RF9 groups relative to CON and FT1RF3 (*p* < 0.05) (Table 5).

### 3.4. Amino Acid Compositions in Fish Tissues

The TAA composition in the liver was not affected by cyclic fasting (Table S1). The free taurine content was lower in all cyclic fasting groups compared to CON, with significantly reduced levels observed in the FT2RF6 and FT3RF9 groups (*p* < 0.05) (Table S2).

Cyclic fasting had minimal effects on the TAA composition in muscle (Table 6). The valine and isoleucine contents in muscle were significantly diminished in the FT3RF9 group compared to all other groups (*p* < 0.05), and were also slightly lower in the FT1RF3 and FT2RF6 groups relative to CON (*p* > 0.05). Analysis of free amino acids revealed that the total free amino acid content in muscle was elevated in all cyclic fasting groups compared to CON, with the magnitude of increase corresponding positively to the duration of single fasting. The branched‐chain amino acid (BCAA) content in muscle was higher in all cyclic fasting groups relative to CON (*p* > 0.05), with isoleucine content being significantly increased in the FT2RF6 and FT3RF9 groups compared to CON (*p* < 0.05). Additionally, the alanine content was significantly higher in the FT2RF6 and FT3RF9 groups than in CON and FT1RF3 (*p* < 0.05), while the alanine content in the FT1RF3 group was slightly lower than in CON (*p* > 0.05) (Table 7).

**Table 6:** Amino acid composition in the muscle of experimental turbot (%, dry matter, mean ± standard error).

Amino acid	CON	FT1RF3	FT2RF6	FT3RF9
Essential amino acids				
Threonine	7.77 ± 0.04	7.65 ± 0.10	7.73 ± 0.08	7.82 ± 0.07
Valine	3.67 ± 0.04^b^	3.53 ± 0.05^b^	3.51 ± 0.07^b^	3.29 ± 0.05^a^
Methionine	2.41 ± 0.07	2.33 ± 0.04	2.15 ± 0.10	2.10 ± 0.15
Isoleucine	3.39 ± 0.03^b^	3.27 ± 0.06^b^	3.21 ± 0.08^b^	2.99 ± 0.05^a^
Leucine	6.22 ± 0.03	6.08 ± 0.08	6.14 ± 0.08	6.15 ± 0.06
Phenylalanine	3.24 ± 0.02	3.12 ± 0.04	3.14 ± 0.04	3.14 ± 0.03
Lysine	7.30 ± 0.04	7.11 ± 0.10	7.21 ± 0.09	7.16 ± 0.07
Histidine	1.61 ± 0.01	1.58 ± 0.02	1.58 ± 0.02	1.55 ± 0.01
Arginine	4.70 ± 0.02	4.58 ± 0.06	4.59 ± 0.03	4.63 ± 0.04
TEAA	36.10 ± 0.25	35.11 ± 0.46	35.09 ± 0.50	34.61 ± 0.36
Nonessential amino acids				
Aspartic acid	1.40 ± 0.02	1.40 ± 0.04	1.43 ± 0.03	1.43 ± 0.04
Serine	3.32 ± 0.01^a^	3.30 ± 0.04^a^	3.38 ± 0.02^ab^	3.47 ± 0.03^b^
Glutamic acid	12.9 ± 0.06	12.6 ± 0.18	12.8 ± 0.12	12.9 ± 0.10
Glycine	3.69 ± 0.05	3.7 ± 0.07	3.65 ± 0.11	3.62 ± 0.10
Alanine	4.62 ± 0.02	4.54 ± 0.04	4.58 ± 0.01	4.65 ± 0.05
Cysteine	1.36 ± 0.07	1.45 ± 0.07	1.42 ± 0.01	1.37 ± 0.05
Tyrosine	2.85 ± 0.03	2.78 ± 0.05	2.79 ± 0.04	2.82 ± 0.03
Proline	2.73 ± 0.03	2.99 ± 0.15	2.91 ± 0.1	2.80 ± 0.06
TNEAA	39.19 ± 0.19	38.98 ± 0.41	39.28 ± 0.13	39.44 ± 0.38
BCAA	13.24 ± 0.10	12.97 ± 0.22	12.82 ± 0.20	12.56 ± 0.19
TAA	76.69 ± 0.41	75.47 ± 0.83	75.80 ± 0.59	75.48 ± 0.71

*Note:* (*n* = 3 replicate tanks; each tank value is the mean of three fish). Within a row, means without a same superscript letter are significantly different (*p* < 0.05).

Abbreviations: BCAAs, branched chain amino acids (isoleucine, leucine, and valine); CON, continuous feeding; EAAs, total essential amino acids; FT1RF3, 1 day fasting followed by 3 days refeeding; FT2RF6, 2 days fasting followed by 6 days refeeding; FT3RF9, 3 days fasting followed by 9 days refeeding; TAAs, total amino acids; TNEAAs, total nonessential amino acids.

**Table 7:** Free amino acid composition in the muscle of experimental turbot (μg/g, dry matter, mean ± standard error).

Amino acid	CON	FT1RF3	FT2RF6	FT3RF9
Taurine acid	11225.36 ± 265.62	11367.56 ± 225.47	11350.77 ± 408.41	11389.76 ± 347.59
Aspartic acid	82.46 ± 2.08^a^	86.86 ± 3.73^a^	86.328 ± 2.83^a^	121.85 ± 8.39^b^
Threonine	434.85 ± 6.01^a^	449.21 ± 11.17^a^	586.86 ± 32.97^b^	639.01 ± 47.04^b^
Serine	964.97 ± 36.80^a^	1211.19 ± 53.97^ab^	1356.54 ± 108.64^b^	1446.12 ± 73.43^b^
Glutamic	1144.85 ± 33.63^a^	1237.08 ± 37.65^ab^	1318.55 ± 37.04^b^	1375.96 ± 39.29^b^
Glycine	2981.51 ± 106	3056.46 ± 161	3091.61 ± 91.39	2834.43 ± 125.92
Alanine	1398.89 ± 36.77^a^	1306.64 ± 34.12^a^	1465.82 ± 81.02^ab^	1664.69 ± 79.78^b^
Cysteine	312.21 ± 7.45^a^	304.71 ± 8.78^a^	325.62 ± 17.89^a^	407.81 ± 24.28^b^
Valine	634.52 ± 63.73	626.26 ± 29.13	742.56 ± 55.70	678.22 ± 40.85
Methionine	50.95 ± 7.05^a^	85.67 ± 10.58^ab^	99.28 ± 9.71^b^	102.64 ± 12.46^b^
Isoleucine	42.62 ± 0.97^a^	48.05 ± 1.10^ab^	69.43 ± 13.70^bc^	73.96 ± 2.74^c^
Leucine	65.77 ± 1.11	72.64 ± 2.52	75.50 ± 10.10	102.09 ± 4.92
Tyrosine	92.97 ± 3.38^a^	90.91 ± 6.13^a^	161.43 ± 27.3^b^	132.35 ± 9.52^ab^
Phenylalanine	104.38 ± 6.34	110.18 ± 8.27	109.01 ± 10.4	104.07 ± 5.49
Lysine	243.25 ± 29.28	284.24 ± 37.97	318.54 ± 46.32	330.33 ± 38.27
Histidine	219.11 ± 11.44	197.07 ± 36.17	231.15 ± 45.05	291.29 ± 30.92
Arginine	339.15 ± 15.09^a^	364.68 ± 31.27^a^	443.46 ± 44.54^ab^	537.47 ± 53.82^b^
Proline	1619.78 ± 84.71	1722.75 ± 219.81	2104.08 ± 266.81	2374.12 ± 299.94
BCAA	706.44 ± 51.44^a^	745.56 ± 31.17^a^	946.58 ± 46.46^b^	868.59 ± 49.32^ab^
TFAA	21732.60 ± 261.79^a^	23353.49 ± 522.12^b^	23590.19 ± 305.53^b^	24422.04 ± 340.69^b^

*Note:* (*n* = 3 replicate tanks; each tank value is the mean of three fish). Within a row, means without a same superscript letter are significantly different (*p* < 0.05).

Abbreviations: BCAAs, branched chain amino acids (isoleucine, leucine, and valine); CON, continuous feeding; FT1RF3, 1 day fasting followed by 3 days refeeding; FT2RF6, 2 days fasting followed by 6 days refeeding; FT3RF9, 3 days fasting followed by 9 days refeeding; TFAAs, total free amino acids.

Cyclic fasting had relatively minor effects on serum free amino acid profiles (Table S3). The glutamate and glycine contents decreased in all cyclic fasting groups, with glutamate content in the FT1RF3 group being significantly reduced compared to CON (*p* < 0.05). The BCAA content showed an increasing trend, and the alanine content in the FT2RF6 and FT3RF9 groups was notably higher than in CON, though the differences did not reach statistical significance (*p* > 0.05).

### 3.5. Biochemical Parameters in the Tissues

#### 3.5.1. Lipid Metabolism‐Related Biochemical Parameters

**Figure 3:**
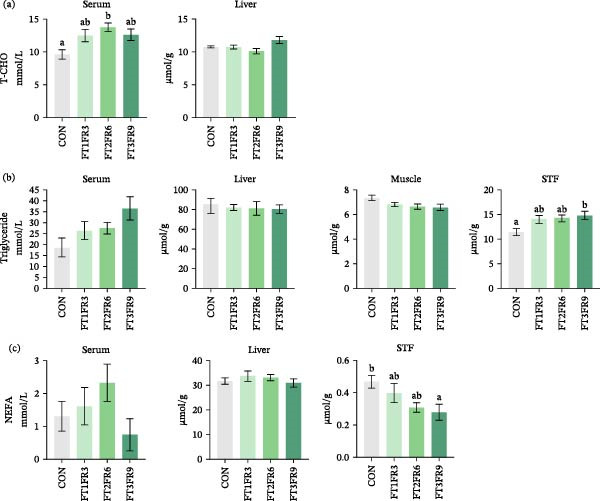
The effects of the experimental feeding regimes on the lipid metabolism‐related biochemical parameters of experimental turbot. (a) Total cholesterol content in serum and liver. (b) Triglyceride content in serum, liver, subcutaneous adipose tissue near the fin (STF) and muscle. (c) Non‐esterified fatty acid (NEFA) content in serum, liver, and STF. All data were reported as mean ± standard error. CON, continuous feeding without fasting; FT1RF3, fasting for 1 day followed by refeeding for 3 days; FT2RF6, fasting for 2 days followed by refeeding for 6 days; FT3RF9, fasting for 3 days followed by refeeding for 9 days (*n* = 3 replicate tanks; each tank value is the mean of three fish). For a certain parameter, the data bars without letter or sharing a same letter are not significantly different (*p* > 0.05).

The serum TG content was elevated in all cyclic fasting groups relative to CON, with the FT3RF9 group showing the highest level (*p* > 0.05) (Figure 3b). The serum T‐CHO content followed a similar pattern, with the FT2RF6 group exhibiting significantly increased levels compared to CON (*p* < 0.05) (Figure 3a). No significant differences in NEFA content were observed among groups (Figure 3c).

The liver and muscle TG content were reduced in all cyclic fasting groups relative to CON (*p* > 0.05), while STF showed an opposite trend, with TG content being significantly elevated in the FT3RF9 group compared to CON (*p* < 0.05) (Figure 3b). Cyclic fasting resulted in decreased NEFA content in STF, with significantly reduced levels observed in the FT2RF6 and FT3RF9 groups relative to CON (*p* < 0.05). No significant differences in the liver NEFA content were observed among cyclic fasting groups (Figure 3c).

#### 3.5.2. Glucose Metabolism‐Related Biochemical Parameters

**Figure 4:**
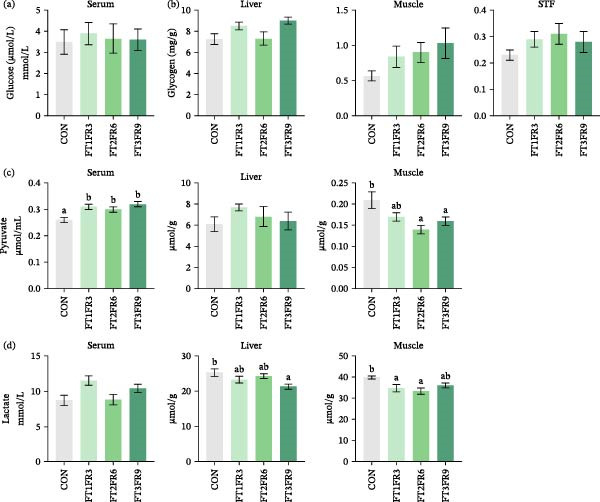
The effects of the experimental feeding regimes on the glucose metabolism‐related biochemical parameters of experimental turbot. (a) Glucose content in serum. (b) Glycogen content in liver, muscle, and subcutaneous adipose tissue near the fin (STF). (c) Pyruvate content in serum, liver, and muscle. (d) Lactate content in serum, liver, and muscle. All data were reported as mean ± standard error. CON, continuous feeding without fasting; FT1RF3, fasting for 1 day followed by refeeding for 3 days; FT2RF6, fasting for 2 days followed by refeeding for 6 days; FT3RF9, fasting for 3 days followed by refeeding for 9 days (*n* = 3 replicate tanks; each tank value is the mean of three fish). For a certain parameter, the data bars without letter or sharing a same letter are not significantly different (*p* > 0.05).

All three cyclic fasting regimens led to elevated blood glucose levels and muscle glycogen accumulation, though the differences were not statistically significant (*p* > 0.05) (Figure 4a,b). The glycogen content in liver and STF showed varying degrees of increase across all cyclic fasting groups, though the effects were relatively modest (*p* > 0.05) (Figure 4b).

The serum pyruvate content was significantly elevated in all cyclic fasting groups compared to CON (*p* < 0.05), and a similar trend was observed in the liver (*p* < 0.05). In contrast, the muscle pyruvate content showed an opposite pattern, being significantly reduced in the FT2RF6 and FT3RF9 groups relative to CON (*p* < 0.05) (Figure 4c).

Regarding LD, cyclic fasting resulted in decreased LD content in both liver and muscle (*p* > 0.05). The liver LD content was significantly reduced in the FT3RF9 group compared to CON (*p* < 0.05), while the muscle LD content was significantly lower in the FT1RF3 and FT2RF6 groups relative to CON (*p* < 0.05). The serum LD content showed varying degrees of increase across all cyclic fasting groups, though the differences were not statistically significant (*p* > 0.05) (Figure 4d).

#### 3.5.3. Amino Acid Metabolism‐Related Biochemical Parameters in Serum

**Figure 5:**
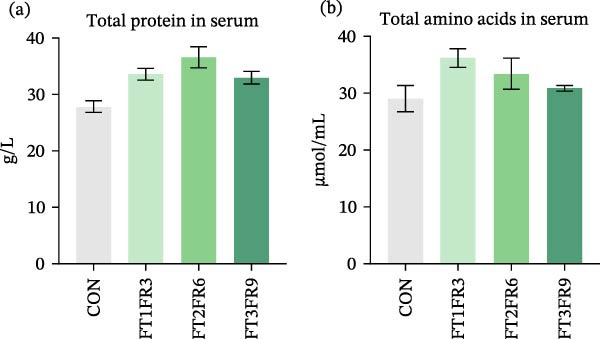
The effects of the experimental feeding regimes on the amino acid metabolism‐related biochemical parameters in the serum of experimental turbot. (a) Total protein content in serum. (b) Total amino acid content in serum. All data were reported as mean ± standard error. CON, continuous feeding without fasting; FT1RF3, fasting for 1 day followed by refeeding for 3 days; FT2RF6, fasting for 2 days followed by refeeding for 6 days; FT3RF9, fasting for 3 days followed by refeeding for 9 days (*n* = 3 replicate tanks; each tank value is the mean of three fish). For a certain parameter, the data bars without letter are not significantly different (*p* > 0.05).

Analysis of serum TP and TAA contents revealed that both parameters were elevated in all cyclic fasting groups compared to CON (*p* > 0.05) (Figure 5a,b).

### 3.6. Transcriptome

#### 3.6.1. Sequence Assembly

Twelve cDNA libraries were sequenced, generating between 40 and 49 million clean reads per library. The Q20 and Q30 values exceeded 98.0% and 95.0%, respectively, and over 90% of clean reads were successfully mapped to the turbot reference genome, indicating high sequencing quality.

#### 3.6.2. Differential Gene Expression Analysis

**Figure 6:**
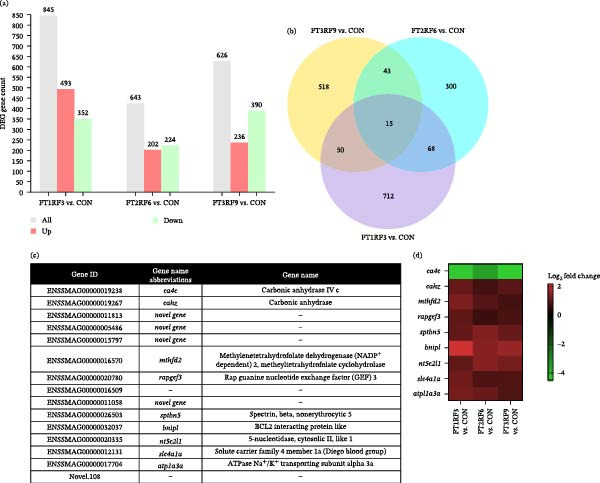
Overview of differentially expressed genes (DEGs) between three fasting regimes and the CON. (a) Bar chart of DEGs between FT1RF3, FT2RF6, and FT3RF9 and the CON, respectively. (b) Venn diagram of DEGs between three fasting modes and the CON. (c) A list of 15 DGEs shared by the three comparisons. (d) Expression changes of nine DGEs with known functions among the 15 DGEs aforementioned. CON, continuous feeding without fasting; FT1RF3, fasting for 1 day followed by refeeding for 3 days; FT2RF6, fasting for 2 days followed by refeeding for 6 days; FT3RF9, fasting for 3 days followed by refeeding for 9 days.

Differential expression analysis (|log_2_FC| > 0, *p*_adj_ < 0.05) revealed 845 DEGs in the comparison of FT1RF3 versus CON (493 upregulated and 352 downregulated), 426 DEGs in FT2RF6 versus CON (202 up and 224 down), and 626 DEGs in FT3RF9 versus CON (236 up and 390 down) (Figure 6a). Among these, 15 DEGs were shared by all three comparisons, of which nine had known functional annotations (Figure 6b–d).

#### 3.6.3. KEGG Enrichment Analysis

**Figure 7:**
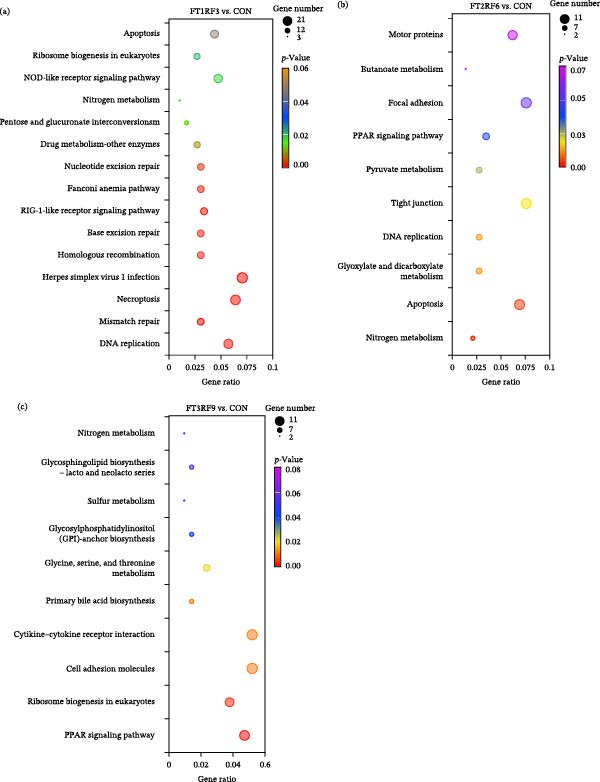
KEGG pathway enrichment analysis of differentially expressed genes (DEGs) between each cyclic fasting group and the control group. Bubble charts show the enriched KEGG pathways for DEGs identified in the comparison of FT1RF3 vs. CON (a), FT2RF6 vs. CON (b), and FT3RF9 vs. CON (c). The *x*‐axis represents the Gene Ratio, calculated as the number of DEGs enriched in a given pathway divided by the total number of annotated genes in that pathway. The *y*‐axis indicates the pathway name. Dot color represents the adjusted *p*‐value (*p*_adj_) after multiple hypothesis testing, with redder colors indicating greater statistical significance. Dot size reflects the number of DEGs enriched in the corresponding pathways.

The KEGG enrichment analysis revealed that DEGs in the FT1RF3 versus CON comparison were significantly enriched in 15 pathways (*p* < 0.05) (Figure 7a, Table S4): DNA replication (dre03030), mismatch repair (dre03430), necroptosis (dre04217), herpes simplex virus 1 infection (dre05168), homologous recombination (dre03440), base excision repair (dre03410), RIG‐I‐like receptor signaling pathway (dre04622), Fanconi anemia pathway (dre03460), nucleotide excision repair (dre03420), drug metabolism‐other enzymes (dre00983), pentose and glucuronate interconversions (dre00040), nitrogen metabolism (dre00910), NOD‐like receptor signaling pathway (dre04621), ribosome biogenesis in eukaryotes (dre03008), and apoptosis (dre04210).

For the comparison of FT2RF6 versus CON, DEGs were significantly enriched in six pathways (*p* < 0.05) (Figure 7b, Table S5): apoptosis (dre04210), glyoxylate and dicarboxylate metabolism (dre00630), DNA replication (dre03030), tight junction (dre04530), pyruvate metabolism (dre00620), and PPAR signaling pathway (dre03320).

For the comparison of FT3RF9 versus CON, DEGs were significantly enriched in eight pathways (*p* < 0.05) (Figure 7c, Table S6): PPAR signaling pathway (dre03320), ribosome biogenesis in eukaryotes (dre03008), cell adhesion molecules (dre04514), cytokine–cytokine receptor interaction (dre04060), primary bile acid biosynthesis (dre00120), glycine, serine and threonine metabolism (dre00260), glycosylphosphatidylinositol (GPI)‐anchor biosynthesis (dre00563), and sulfur metabolism (dre00920).

#### 3.6.4. Expression Patterns of Genes Related to Metabolic Pathways

Given the observed changes in body composition across cyclic fasting groups, DEGs involved in glucose, lipid, and amino acid metabolism, as well as mitochondrial respiratory chain complexes, were further analyzed. A total of 41 DEGs were identified, including eight glucose metabolism‐related genes (glycogen synthesis: *gys*; glycogen breakdown: *pygl*; glycolysis: *pfkp*, *pklr*; gluconeogenesis: *pck2*, *fbp1a*, *g6pc3*; pentose phosphate pathway: *g6pdh*), nine lipid metabolism‐related genes (lipogenesis: *fasn*, *acaca*, *scd*; lipolysis: *lipea*, *mgll*, *CD36*, *pparα*; fatty acid β‐oxidation: *cpt2*, *acox1*), eight amino acid metabolism‐related genes (promoting protein degradation: glud1b, *glsa*, *gls2b*, *dbt*; inhibiting protein synthesis: *gcn1*; promoting protein synthesis: *rptor*, *igf1*, and *rps6kb1b*), and 16 mitochondrial respiratory chain complex genes (*nd1*, *nd2*, *nd3*, *nd4*, *nd4l*, *nd5*, *nd6*, *sdhc*, *uqcrc*2, *cyc1*, *cytb*, *cox5b2*, *cox5a*, *cox1*, *cox2*, and *cox3*).

**Figure 8:**
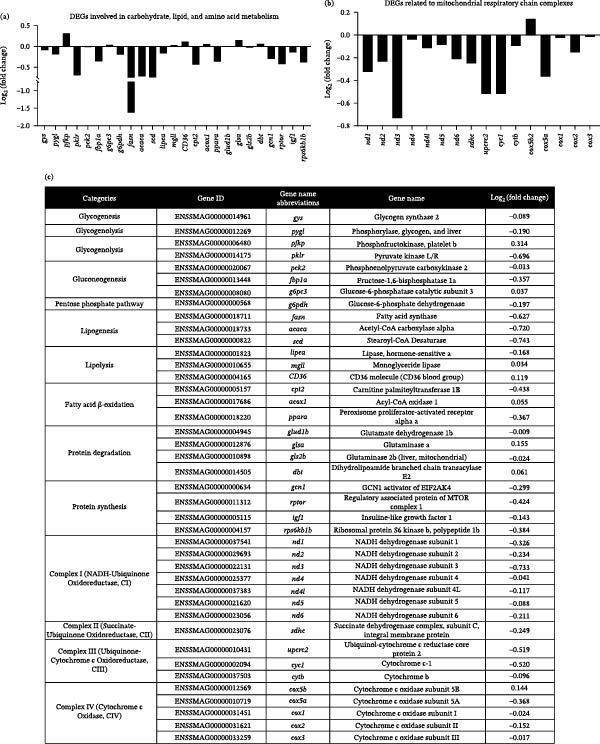
Differentially expressed genes (DEGs) in carbohydrate metabolism, lipid, amino acid metabolism, and mitochondrial respiratory chain complexes (FT1RF3 vs. CON). (a) DEGs involved in carbohydrate, lipid, and amino acid metabolism. (b) DEGs related to mitochondrial respiratory chain complexes. (c) List of DEGs. CON, continuous feeding, FT1RF3, fasting for 1 day followed by refeeding for 3 days (*n* = 3).

The expression patterns of these genes varied across treatments. In the FT1RF3 group, upregulated genes included *pfkp*, *fbp1a*, *mgll*, *CD36*, *acox1*, *glsa*, *dbt*, and *cox5b2*, while downregulated genes included *gys*, *pygl*, *pklr*, *pck2*, *g6pc3*, *g6pdh*, *fasn*, *acaca*, *scd*, *lipea*, *cpt2*, *pparα*, *nd1*, *nd2*, *nd3*, *nd4*, *nd4l*, *nd5*, *nd6*, *sdhc*, *uqcrc2*, *cyc1*, *cytb*, *cox5a*, *cox1*, *cox2*, *cox3*, *glud1b*, *gcn1*, *rptor*, *igf1*, and *rps6kb1b* (Figure 8).

**Figure 9:**
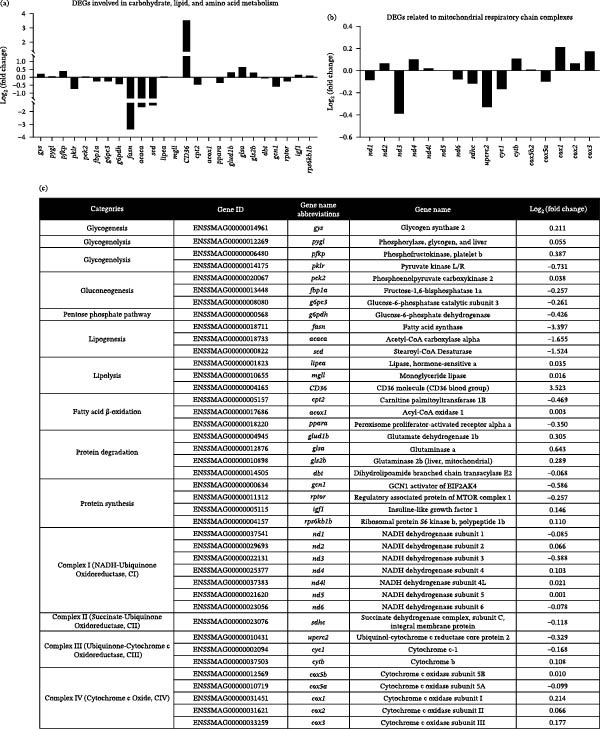
Differentially expressed genes (DEGs) in carbohydrate metabolism, lipid, amino acid metabolism, and mitochondrial respiratory chain complexes (FT2RF6 vs. CON). (a) DEGs involved in carbohydrate, lipid, and amino acid metabolism. (b) DEGs related to mitochondrial respiratory chain complexes. (c) List of DEGs. CON, continuous feeding; FT2RF6, fasting for 2 days followed by refeeding for 6 days (*n* = 3).

In the FT2RF6 group, upregulated genes included *gys*, *pygl*, *pfkp*, *CD36*, *glud1b*, *glsa*, *gls2b*, *nd2*, *nd4*, *cytb*, *cox1*, *cox2*, and *cox3*, while downregulated genes included *pklr*, *fbp1a*, *g6pc3*, *g6pdh*, *fasn*, *acaca*, *scd*, *pparα*, *cpt2*, *gcn1*, and *rptor* (Figure 9).

**Figure 10:**
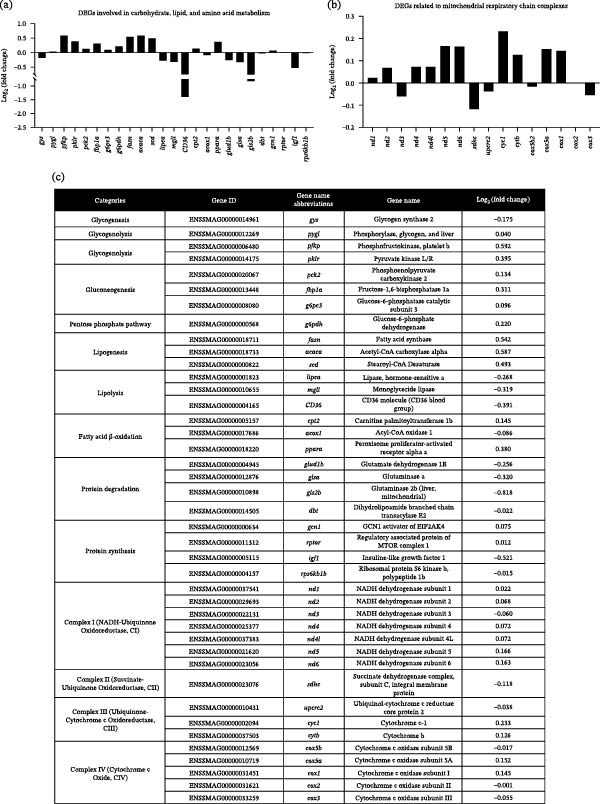
Differentially expressed genes (DEGs) in carbohydrate metabolism, lipid, amino acid metabolism, and mitochondrial respiratory chain complexes (FT3RF9 vs. CON). (a) DEGs involved in carbohydrate, lipid, and amino acid metabolism. (b) DEGs related to mitochondrial respiratory chain complexes. (c) List of DEGs. CON, continuous feeding; FT3RF9, fasting for 3 days followed by refeeding for 9 days (*n* = 3).

In the FT3RF9 group, upregulated genes included *fbp1a*, *g6pc3*, *g6pdh*, *fasn*, *acaca*, *scd*, *cpt2*, *pparα*, *nd1*, *nd2*, *nd4*, *nd4l*, *nd5*, *nd6*, *cyc1*, *cytb*, *cox5a*, *cox1*, *cox2*, and *gcn1*, while downregulated genes included *gys*, *lipea*, *mgll*, *CD36*, *acox1*, *glud1b*, *glsa*, *gls2b*, *igf1*, *nd3*, *sdhc*, *uqcrc2*, *cox5b2*, and *cox3* (Figure 10).

## 4. Discussion

Strategies that reduce feed input while maintaining growth performance offer a promising approach to lower economic costs and minimize feed waste. In the present study, fish in the FT1RF3 group achieved a final body weight comparable to that of the CON, suggesting a potential for compensatory growth, and exhibited a numerically lower FCR relative to that of CON, though the difference was not statistically significant. In contrast, the FT2RF6 and FT3RF9 groups consistently exhibited numerically lower final body weights than that of CON, suggesting a tendency toward growth suppression over the 72‐day period. These findings are consistent with previous reports demonstrating that appropriately designed fasting strategies can effectively promote growth while reducing feed consumption, thereby improving aquaculture efficiency [9]. However, when the fasting duration exceeds the species‐specific tolerance threshold, energy is preferentially allocated to metabolic maintenance rather than somatic growth [4, 15, 19]. Similar observations have been reported in longfin yellowtail *Seriola rivoliana*, where compensatory growth was not observed under fasting cycles of 3–7 days, followed by 7 days of refeeding over 85 days. In contrast, partial compensatory growth was achieved when the same species was subjected to milder cycles of 2 days of fasting and 1 day of refeeding for 89 days [13, 14]. These findings indicate that when the fasting cycles exceed a critical duration, the fasting stress becomes too severe to elicit compensatory growth. The difficulty in achieving compensation effects under prolonged or severe feed restriction may be attributed to fish reaching a “point of no return.” When organisms experience excessive growth stunting or exhibit alterations in physiological functions, the compensatory growth response may be constrained [20–23].

In the present study, different cyclic fasting regimens were associated with diametrically opposite lipid metabolic responses, suggesting that the duration of a single fasting event serves as a threshold determining the shift in the metabolic strategy. In the FT1RF3 group, the liver lipid content was significantly higher than that of CON, and the muscle lipid content was numerically higher, while the STF lipid content tended to be lower. Concurrently, the serum TG and liver NEFA levels were higher than those in the CON, whereas the STF NEFA content was significantly reduced (*p* < 0.05). These results indicate that moderate fasting pressure activated adipose tissue mobilization, releasing NEFA for utilization by peripheral tissues. This finding was highly consistent with a previous study on turbot, which reported that 30 days of continuous fasting led to a linear decrease in lipid content in the liver and STF but a linear increase in muscle lipid content, suggesting a directional transfer of lipids from storage tissues (STF and liver) to utilizing tissues (muscle) to support fatty acid β‐oxidation [24]. In contrast, the two cyclic fasting groups with single fasting durations exceeding 1 day (FT2RF6 and FT3RF9) exhibited completely opposite metabolic phenotypes: lipid accumulation in the whole body, liver, muscle, and STF, with no significant changes in the liver NEFA content and significantly lower STF NEFA content compared to the CON. These characteristics indicate that when the single fasting duration exceeded the tolerance threshold of 1 day, the metabolic strategy of juvenile turbot in this study appeared to shift toward reserve prioritization to cope with the risk of potential future food scarcity.

This survival‐oriented metabolic reprograming represents an evolutionarily conserved adaptive strategy across animal taxa. When feed restriction is too severe or prolonged, recovery of energy reserves is often prioritized over growth to prevent metabolic exhaustion and ensure survival [9]. Studies on red‐claw crayfish (*Cherax quadricarinatus*) found that juveniles subjected to 45 days of cyclic fasting (4 days of fasting and 4 days of refeeding) did not achieve compensatory growth, but the hepatopancreatic lipid content fully recovered to control levels [25]. Similar phenomena were observed in gilthead seabream (*Sparus aurata*) juveniles: after 3 weeks of fasting followed by 7 weeks of refeeding, although compensatory growth was not triggered, the whole‐body lipid and energy content fully recovered to control levels [26]. In rohu (*Labeo rohita*) subjected to 1, 2, or 3 weeks of fasting, followed by 2 weeks of refeeding, the body protein and lipids were fully restored, but only individuals fasted for 1–2 weeks achieved complete compensatory growth [27]. Similarly, studies on Atlantic halibut (*Hippoglossus hippoglossus*), Chinese sturgeon (*Acipenser sinensis*), and black rockfish (*Sebastes schlegelii*) have all confirmed that when fasting pressure exceeds a threshold, energy reserve recovery becomes the primary metabolic target of the organism, even at the expense of growth [20, 28, 29]. This provides an explanation for the lipid accumulation and growth suppression observed in the FT2RF6 and FT3RF9 groups in the present study.

Regarding the effects of cyclic fasting on fatty acid composition, although previous studies have demonstrated tissue‐specific preferences in fatty acid mobilization in turbot [24], the three cyclic fasting regimens in the present study had relatively mild effects on the tissue fatty acid profiles. This observation may be attributed to the strong influence of diets on tissue fatty acid composition in farmed fish [30] as all treatment groups in the present study received the same commercial diet during refeeding.

In all cyclic fasting groups, elevated liver pyruvate content and blood glucose levels were observed, along with decreased liver LD content. The LD is the end product of anaerobic glycolysis; its decrease coupled with increased pyruvate suggests the inhibition of the anaerobic glycolytic pathway, with energy metabolism relying primarily on aerobic oxidation. The muscle glycogen content increased while the pyruvate and LD levels decreased. This pattern was consistent with the metabolic adaptive strategies of teleost fish under feed‐restricted conditions: organisms can synthesize glucose through gluconeogenesis, using noncarbohydrate precursors such as LD and glycerol, to maintain blood glucose homeostasis and support tissue glycogen stores [31–35].

Notably, the protein content in the whole body and various tissues remained unaffected across all cyclic‐fasting groups. Protein is the last energy reserve to be mobilized during fasting in fish, and only after substantial depletion of lipid reserves does the organism initiate muscle protein catabolism for energy [8]. The observed changes in lipid metabolism in this study aligned with this hierarchical pattern of energy reserve utilization.

The nine annotated DEGs shared by different intergroup comparisons identified in this study provide molecular insights into how cyclic fasting reshapes liver metabolism in juvenile turbot. These transcriptional changes align with the evolutionarily conserved adaptive responses to intermittent fasting proposed by de Cabo and Mattson [36]. Downregulation of *ca4c* suggested reduced metabolic demand, while upregulation of *nt5c2l1* and *mthfd2* reflected enhanced nucleotide recycling and one‐carbon metabolism, key strategies for energy optimization under nutrient‐restricted conditions [37, 38]. Cyclic fasting also bolstered cellular defense mechanisms, as evidenced by increased expression of *bnipl* (apoptosis‐related) and *sptbn5* (cytoskeletal stability), supporting the concept of fasting‐induced stress resistance [39, 40]. Concurrent upregulation of ion transporters (*cahz*, *slc4a1a*, and *atp1a3a*) and the signaling mediator *rapgef3* further illustrated a coordinated effort to maintain homeostasis and orchestrate adaptive responses [41, 42]. These findings suggest that cyclic fasting elicits a shared liver adaptive program in juvenile turbot, characterized by energy conservation, molecular recycling, and cytoprotection.

The KEGG enrichment analysis further validated these transcriptional changes, revealing pathway‐level reprograming that varied with fasting duration. Under the present experimental conditions, although all three regimens exhibited a common signature of energy conservation, the duration of the single fasting event dictated distinct metabolic and cellular strategies.

Under the shortest fasting duration (FT1RF3), fish exhibited an adaptive phenotype characterized by comprehensive metabolic suppression and enhanced cellular defense. DEGs were significantly enriched in pathways, including DNA replication (dre03030), mismatch repair (dre03430), and necroptosis (dre04217). Genes positively regulating DNA replication and repair pathways, including those involved in DNA replication, mismatch repair, homologous recombination, base excision repair, and the Fanconi anemia pathway were uniformly downregulated (*mcm3*, *mcm4*, *mcm5*, *mcm6*, *mcm7*, *pold1*, *pole*, *pola1*, and *lig1*) (Table S4), indicating reduced replicative activity and lower error tolerance during DNA synthesis [43–47]. Concurrently, the upregulation of *stat1*, *stat2*, and *stat3*, key transcriptional regulators of necroptosis [48], along with the induction of *Caspase10*, an initiator caspase in the death receptor‐mediated apoptotic pathway [49, 50], collectively activated both necroptosis and apoptosis programs (Table S4). This coordinated response suggested the active clearance of suboptimal cells to optimize tissue function. Enhanced expression of *irf3*, *irf7*, *dhx58*, and *trim25* indicated activation of NOD‐like receptor and RIG‐I‐like receptor signaling pathways, reflecting heightened innate immune surveillance in response to potential damage signals [51–53] (Table S4). Downregulation of *dcxr* and *xylb* in the pentose and glucuronate interconversion pathway (dre00040) further supported reduced nucleotide synthesis and NADPH regeneration, reinforcing the suppression of DNA repair and cell proliferation [54, 55] (Table S4). FT1RF3 was also associated with the broad suppression of energy metabolism. Rather than indicating metabolic impairment, this suppression, together with the reduced DNA replication and repair activities observed, likely represents a coordinated energy‐saving strategy that prioritizes resource allocation to processes essential for compensatory growth. Genes involved in glycogen synthesis (*gys*) and breakdown (*pygl*), glycolysis (*pklr*), gluconeogenesis (*pck2*, *fbp1a*, and *g6pc3*), lipid synthesis (*fasn, acaca*, and *scd*), lipid breakdown (*lipea*, *cpt2*, and *pparα*), and protein synthesis (*rptor*, *igf1*, and *rps6kb1b*) were all downregulated. Key genes encoding mitochondrial respiratory chain complexes (*nd1*, *nd2*, *nd3*, *nd4*, *nd4l*, *nd5*, *nd6*, *sdhc*, *uqcrc2*, *cyc1*, *cytb*, *cox1*, *cox2*, *cox3*, and *cox5a*) and TCA cycle rate‐limiting enzymes (*mdh1 and cs*) were also suppressed (Table S4). These changes aligned with reduced body lipid and accumulation of BCAAs in the liver and muscle. Notably, while *pklr* was downregulated, *pfkp* was upregulated, and despite elevated pyruvate levels in serum and liver, *cs* expression decreased, suggesting that glycolysis was active but subject to feedback inhibition due to pyruvate accumulation. Upregulation of *mgll*, *CD36*, and *acox1* pointed to a shift toward utilizing free fatty acids as the primary energy source under low metabolic demand. These responses, through energy conservation, reduced unnecessary proliferation, enhanced immune preparedness, and promotion of cellular renewal and tissue homeostasis, may collectively provide the metabolic basis for the compensatory growth potential observed in this group.

Under the intermediate fasting duration (FT2RF6), a more conservative survival‐oriented strategy emerged. The DNA replication remained suppressed (*mcm5*, *mcm6*, and *pole*), but the apoptosis was inhibited (downregulation of *atm*, *pidd1*, and *capn1b*), suggesting that cell survival was prioritized over tissue optimization, consistent with the reduced weight gain observed in this group [49, 56–58] (Table S5). Glycolysis (*pfkp* and *pygl*) and glycogen synthesis (*gys*) were enhanced, while gluconeogenesis was suppressed (*pck2*, *fbp1a*, and *g6pc3*), indicating preferential utilization of existing glycogen stores. Within the PPAR signaling pathway, downregulation of *scd* and *acsl3* reflected reduced fatty acid desaturation and activation, while suppression of *fasn* and *acaca* indicated decreased de novo lipogenesis. However, enhanced fatty acid uptake (*CD36* upregulation), coupled with reduced fatty acid oxidation (*cpt2*) and TCA cycle efficiency (*mdh1* and *cs*), may have contributed to lipid accumulation in the liver, muscle, and whole body [59]. Amino acid catabolism was upregulated (*glud1b*, *glsa*, and *gls2b*), while protein synthesis was suppressed (*rptor* downregulation), which may account for the accumulation of free BCAAs and glutamate in muscle and elevated TAAs in serum. These results suggested that cyclic fasting regimens with single fasting durations of 2 days (FT2RF6) or more shift juvenile turbot toward survival‐oriented strategies that prioritize energy storage at the expense of growth and tissue remodeling.

Under the longest fasting duration (FT3RF9), an extreme reserve‐oriented phenotype was observed. Within the PPAR signaling pathway, genes promoting fatty acid synthesis were uniformly upregulated (*acsl4a*, *fads2*, *fasn*, *acaca*, and *scd*), while lipid uptake (*CD36*) and breakdown (*lpla*, *lipea*, and *mgll*) were suppressed, together driving lipid accumulation in the liver, muscle, and whole body [60–62]. Glycogen breakdown (*pygl*), glycolysis (*pfkp* and *pklr*), gluconeogenesis (*pck2*, *fbp1a*, and *g6pc3*), and the pentose phosphate pathway (*g6pdh*) were all enhanced, likely providing precursors and NADPH for lipid synthesis [63]. Although amino acid catabolism genes (*glud1b*, *glsa*, *gls2b*, and *dbt*) were downregulated, serum and muscle free BCAA levels increased, suggesting free amino acids were held in reserve for potential future energy needs. While key regulators of protein synthesis (*rptor* and *rps6kb1b*) showed no change, eight genes positively regulating ribosome biogenesis (*nop56*, *gnl2*, *afg2a*, *utp4*, and *rrp7a*) were downregulated, implying impaired ribosomal assembly and protein synthesis capacity, consistent with *igf1* downregulation and reduced growth [64] (Table S6). Downregulation of tight junction proteins (*cldn15la*, *cldn11a*, and *cldn33b*) within the cell adhesion molecule pathway further suggested compromised liver integrity [65]. These findings indicate that prolonged fasting (a single 3‐day fasting) redirects resources toward energy reserves, potentially at the cost of growth and tissue structure maintenance.

## 5. Conclusions

Under the present experimental conditions, the 1‐day fasting/3‐day refeeding regimen (FT1RF3) achieved growth comparable to that of continuously fed controls while numerically improving feed conversion. The result was accompanied by a coordinated metabolic adaptation characterized by broad metabolic suppression, enhanced fatty acid utilization, stimulated innate immunity, and promotion of cellular renewal, as revealed by transcriptomic analysis. This adaptation may help balance maintenance metabolism with somatic growth. In contrast, extending single fasting events to 2–3 days promoted lipid accumulation through transcriptional suppression of fatty acid oxidation, reflecting a shift toward a survival‐oriented metabolic strategy at the expense of growth. Notably, the whole‐body protein, amino acid, and fatty acid profiles were largely conserved across regimens, suggesting that the nutritional composition of the fish was not compromised. However, validation of the transcriptomic findings at the transcript and protein levels, together with evaluation of full dynamic changes throughout the fasting–refeeding cycles, would help substantiate the proposed mechanisms. These findings indicate that the fasting episode length is a key determinant of divergent metabolic trajectories in juvenile turbo. It should be noted; however, that sensitivity to fasting may differ across developmental stages; therefore, the applicability of the FT1RF3 regimen to other growth phases or production settings requires context‐specific evaluation.

## Funding

This work was supported by the National Key R&D Program of China (Grant 2024YFD2402000), the State Key Laboratory of Mariculture Biobreeding and Sustainable Goods (Grant BRESG202405), the Taishan Scholars Program, Chinese Academy of Fishery Sciences (Grants 2025XT0804 and 2023TD52), and the China Agricultural Research System (Grant CARS‐47).

## Ethics Statement

Fish management and sampling protocols in this experiment were authorized by the Animal Experimental Ethical Inspection of the Animal Care and Use Committee of the Yellow Sea Fisheries Research Institute (Number ACUC202309280529), ensuring no novel interventions that could cause animal harm or distress were used.

## Conflicts of Interest

The authors declare no conflicts of interest.

## Supporting Information

Additional supporting information can be found online in the Supporting Information section.

## Supporting information


**Supporting Information** Table S1: Amino acid composition in the liver of experimental turbot (% dry matter, mean ± standard error). Table S2: Free amino acid composition in the liver of experimental turbot (μg/g dry matter, mean ± standard error). Table S3: Free amino acid composition in the serum of experimental turbot (μg/g dry matter, mean ± standard error). Table S4: Differentially expressed genes (DEGs) between FT1RF3 and CON in significantly enriched KEGG pathways (*p*‐value < 0.05). Table S5: Differentially expressed genes (DEGs) between FT2RF6 and CON in significantly enriched KEGG pathways (*p*‐value < 0.05). Table S6: Differentially expressed genes (DEGs) between FT3RF9 and CON in significantly enriched KEGG pathways (*p*‐value < 0.05).

## Data Availability

The sequencing data obtained from the transcriptomic analysis were deposited into GenBank of NCBI under the accession number PRJNA1434104. All other data supporting the findings of this study are available within the paper and its Supporting Information.
