# Supplementary material for: Metabolic Responses to Cyclic Fasting in Juvenile Turbot (Scophthalmus maximus)
Source: Aquac Nutr. 2026 Jun 25;2026:7599690. doi: 10.1155/anu/7599690 (PMC13305143; doi:10.1155/anu/7599690)
Supplement: Supplementary file 1 — Supporting Information Table S1: Amino acid composition in the liver of experimental turbot (% dry matter, mean ± standard error). Table S2: Free amino acid composition in the liver of experimental turbot (μg/g dry matter, mean ± standard error). Table S3: Free amino acid composition in the serum of experimental turbot (μg/g dry matter, mean ± standard error). Table S4: Differentially expressed genes (DEGs) between FT1RF3 and CON in significantly enriched KEGG pathways (p‐value < 0.05). Table S5: Differentially expressed genes (DEGs) between FT2RF6 and CON in significantly enriched KEGG pathways (p‐value < 0.05). Table S6: Differentially expressed genes (DEGs) between FT3RF9 and CON in significantly enriched KEGG pathways (p‐value < 0.05). [file ANU-2026-7599690-s001.docx]

**Supplementary Table S1** Amino acid composition in the liver of experimental turbot (% dry matter, mean ± standard error).

| Amino acid | CON | FT1RF3 | FT2RF6 | FT3RF9 |
| --- | --- | --- | --- | --- |
| Essential amino acids | | | | |
| Threonine | 1.26±0.03 | 1.20±0.06 | 1.40±0.08 | 1.20±0.06 |
| Valine | 1.39±0.04 | 1.37±0.06 | 1.49±0.08 | 1.37±0.05 |
| Methionine | 0.49±0.07 | 0.48±0.06 | 0.59±0.04 | 0.59±0.07 |
| Isoleucine | 1.07±0.03 | 1.06±0.04 | 1.18±0.06 | 1.13±0.08 |
| Leucine | 2.02±0.06 | 2.00±0.09 | 2.22±0.12 | 2.06±0.11 |
| Phenylalanine | 1.21±0.03 | 1.22±0.05 | 1.35±0.07 | 1.20±0.06 |
| Lysine | 2.07±0.06 | 2.07±0.09 | 2.29±0.12 | 2.02±0.08 |
| Histidine | 0.67±0.02 | 0.69±0.04 | 0.73±0.04 | 0.72±0.03 |
| Argnine | 1.59±0.05 | 1.53±0.09 | 1.73±0.09 | 1.54±0.10 |
| TEAA | 11.77±0.35 | 11.63±0.49 | 12.98±0.68 | 12.1±0.68 |
| Non-essential amino acids | | | | |
| Aspartic acid | 2.42±0.06 | 2.32±0.12 | 2.69±0.17 | 2.42±0.14 |
| Serine | 1.25±0.03 | 1.20±0.07 | 1.33±0.07 | 1.19±0.06 |
| Glutamic acid | 3.64±0.10 | 3.55±0.17 | 3.96±0.17 | 3.54±0.15 |
| Glycine | 1.28±0.04 | 1.29±0.08 | 1.34±0.05 | 1.27±0.06 |
| Alanine | 1.66±0.04 | 1.61±0.08 | 1.81±0.09 | 1.67±0.06 |
| Cysteine | 0.78±0.05 | 0.85±0.03 | 0.96±0.04 | 1.03±0.12 |
| Tyrosine | 0.91±0.03 | 0.87±0.05 | 1.04±0.05 | 1.02±0.08 |
| Proline | 1.01±0.02 | 0.95±0.07 | 1.18±0.13 | 1.03±0.03 |
| TNEAA | 12.96±0.36 | 12.66±0.63 | 14.33±0.67 | 13.41±0.70 |
| BCAA | 4.49±0.13 | 4.43±0.19 | 4.89±0.26 | 4.51±0.26 |
| TAA | 24.73±0.71 | 23.93±1.18 | 27.3±1.35 | 24.93±1.14 |

EAA = total essential amino acids, TNEAA = total non-essential amino acids; BCAA = branched chain amino acids (isoleucine, leucine, and valine); TAA = total amino acids.

CON, continuous feeding; FT1RF3, 1 d fasting followed by 3 d refeeding; FT2RF6, 2 d fasting followed by 6 d refeeding; FT3RF9, 3 d fasting followed by 9 d refeeding (n=3 replicate tanks; each tank value is the mean of 3 fish). Within a row, means without a same superscript letter are significantly different (*P* < 0.05).

**Supplementary Table S2** Free amino acid composition in the liver of experimental turbot (μg/g dry matter, mean ± standard error).

| Amino acid | CON | FT1RF3 | FT2RF6 | FT3RF9 |
| --- | --- | --- | --- | --- |
| Taurine acid | 6497.01±211.58^b^ | 5739.86±268.67^ab^ | 5587.71±219.69^a^ | 5513.76±163.01^a^ |
| Aspartic acid | 310.6±84.8 | 390.64±130.66 | 474.27±84.46 | 419.07±87.75 |
| Threonine | 630.34±55.65 | 670.94±54.72 | 719.89±41.24 | 529.38±58.49 |
| Serine | 513.56±44.21 | 546.33±56.97 | 590.58±39.4 | 481.87±57.2 |
| Glutamic | 2821.24±146.77 | 2944.12±76.7 | 2987.73±200.77 | 2582.62±155.24 |
| Glycine | 555.91±41.49 | 549.21±36.86 | 575.39±37.42 | 541.65±50.79 |
| Alanine | 3351.1±134.79 | 3234.05±148.3 | 3571.78±140.01 | 3138.79±108.7 |
| Cysteine | 423.43±52.96 | 432.23±53.93 | 488.77±44.98 | 397.67±48.66 |
| Valine | 394.66±46.23 | 423.35±62.45 | 493.96±45.44 | 438.1±54.78 |
| Methionine | 249.04±41.61 | 259.99±55.03 | 331.34±22.92 | 244.71±42.39 |
| Isoleucine | 277.4±38.73 | 301.67±52.75 | 348.72±32.81 | 301.7±45.36 |
| Leucine | 627.68±103.78 | 691.08±136.61 | 800.14±78.28 | 674.37±111.13 |
| Tyrosine | 256.2±29.43 | 290.08±41.85 | 323.11±28.01 | 259.75±30.81 |
| Phenylalanine | 415.00±49.39 | 432.38±62.02 | 470.19±40.37 | 398.18±57.7 |
| Lysine | 474.08±65.9 | 517.08±89.86 | 601.1±59.22 | 508.99±78.37 |
| Histidine | 340.87±26.12 | 318.96±13.89 | 341.56±23.13 | 291.04±19.09 |
| Argnine | 488.99±60.89 | 549.77±79.7 | 611.76±43.53 | 456.15±67.67 |
| Proline | 394.94±30.36 | 400.89±29.41 | 450.88±35.09 | 390.48±45.57 |
| BCAA | 1300±188 | 1416±251 | 1643±152 | 1414±211 |
| TFAA | 1299.75±188.1 | 1416.1±251.04 | 1642.82±152.42 | 1414.17±210.52 |

BCAA = branched chain amino acids (isoleucine, leucine, and valine); TFAA = total free amino acids.

CON, continuous feeding; FT1RF3, 1 d fasting followed by 3 d refeeding; FT2RF6, 2 d fasting followed by 6 d refeeding; FT3RF9, 3 d fasting followed by 9 d refeeding (n=3 replicate tanks; each tank value is the mean of 3 fish). Within a row, means without a same superscript letter are significantly different (*P* < 0.05).

**Supplementary Table S3** Free amino acid composition in the serum of experimental turbot (μg/g dry matter, mean ± standard error).

| Amino acid | CON | FT1RF3 | FT2RF6 | FT3RF9 |
| --- | --- | --- | --- | --- |
| Taurine acid | 44.01±2.55 | 44.01±2.55 | 44.01±2.55 | 44.01±2.55 |
| Aspartic acid | 7.90±0.60 | 6.40±0.10 | 7.10±0.20 | 7.20±0.00 |
| Threonine | 13.42±1.66 | 11.17±0.41 | 14.35±0.61 | 14.67±0.46 |
| Serine | 10.32±1.27 | 8.63±0.12 | 11.92±0.48 | 11.01±0.81 |
| Glutamic | 12.38±2.28 | 7.67±0.76 | 8.67±0.32 | 8.85±1.08 |
| Glycine | 9.60±1.10^b^ | 5.60±0.30^a^ | 7.10±0.20^ab^ | 6.60±0.60^ab^ |
| Alanine | 36.81±2.63 | 35.78±2.1 | 43.28±0.69 | 42.92±1.76 |
| Cysteine | 9.00±2.56 | 10.2±1.49 | 11.24±0.3 | 14.39±1.47 |
| Valine | 14.22±14.22 | 13.92±13.92 | 17.88±17.88 | 18.35±18.35 |
| Methionine | 19.59±3.83 | 20.19±4.74 | 20.4±4.6 | 16.2±1.32 |
| Isoleucine | 6.42±0.73 | 8.69±0.89 | 8.76±1.57 | 10.72±2.17 |
| Leucine | 12.18±0.27 | 14.19±0.59 | 15.44±2.47 | 16.88±3.35 |
| Tyrosine | 14.00±0.88 | 14.36±0.88 | 16.57±3.64 | 15.77±1.7 |
| Phenylalanine | 14.38±0.47 | 15.97±0.51 | 16.84±1.91 | 16.92±2.18 |
| Lysine | 24.76±0.59 | 26.13±1.28 | 29.86±0.77 | 25.02±3.47 |
| Histidine | 8.84±0.81 | 9.11±0.25 | 8.36±0.49 | 8.00±0.69 |
| Argnine | 12.9±0.78 | 12.96±0.48 | 15.67±0.31 | 14.88±0.97 |
| Proline | 6.36±0.47 | 6.42±1.24 | 6.71±0.71 | 5.67±0.45 |
| BCAA | 32.82±0.91 | 36.80±1.99 | 42.08±7.04 | 45.95±8.81 |
| TFAA | 277.13±10.55 | 272.05±16.94 | 305.46±18.68 | 298.55±11.64 |

BCAA = branched chain amino acids (isoleucine, leucine, and valine); TFAA = total free amino acids.

CON, continuous feeding; FT1RF3, 1 d fasting followed by 3 d refeeding; FT2RF6, 2 d fasting followed by 6 d refeeding; FT3RF9, 3 d fasting followed by 9 d refeeding (n=3 replicate tanks; each tank value is the mean of 3 fish). Within a row, means without a same superscript letter are significantly different (*P* < 0.05).

**Supplementary Table S4** Differentially expressed genes (DEGs) between FT1RF3 and CON in significantly enriched KEGG pathways (*p-value* < 0.05).

| Gene | Description | Log_2_FC | *P*-value | KEGG Pathway |
| --- | --- | --- | --- | --- |
| *mcm6* | Minichromosome maintenance complex component 6 | -1.370 | 2.09E-04 | DNA replication |
| *mcm3* | Minichromosome maintenance complex component 3 | -1.498 | 4.83E-04 | DNA replication |
| *mcm4* | Minichromosome maintenance complex component 4 | -1.457 | 2.87E-03 | DNA replication |
| *pold1* | Polymerase (DNA directed), delta 1, catalytic subunit | -1.133 | 3.37E-03 | DNA replication; Mismatch repair; Homologous recombination; Base excision repair; Nucleotide excision repair |
| *pole* | Polymerase (DNA directed), epsilon | -1.337 | 6.14E-03 | DNA replication; Base excision repair; Nucleotide excision repair |
| *pola1* | Polymerase (DNA directed), alpha 1 | -0.766 | 1.32E-02 | DNA replication |
| *mcm5* | Minichromosome maintenance complex component 5 | -1.247 | 1.44E-02 | DNA replication |
| *rpa1* | Replication protein A1 | -0.800 | 2.10E-02 | DNA replication; Mismatch repair; Homologous recombination; Fanconi anemia pathway; Nucleotide excision repair |
| *lig1* | Ligase I, DNA, ATP-dependent | -0.677 | 2.77E-02 | DNA replication; Mismatch repair; Base excision repair; Nucleotide excision repair |
| *mlh1* | MutL homolog 1, colon cancer, nonpolyposis type 2 (E. coli) | -0.684 | 2.21E-02 | Mismatch repair; Fanconi anemia pathway |
| *stat1a* | Signal transducer and activator of transcription 1a | 1.206 | 1.11E-03 | Necroptosis; Herpes simplex virus 1 infection; NOD-like receptor signaling pathway |
| *casp10* | Caspase 10, apoptosis-related cysteine peptidase | 1.387 | 1.35E-03 | Necroptosis; Herpes simplex virus 1 infection; RIG-I-like receptor signaling pathway; NOD-like receptor signaling pathway; Apoptosis |
| *stat2* | Signal transducer and activator of transcription 2 | 0.951 | 1.44E-03 | Necroptosis; Herpes simplex virus 1 infection; NOD-like receptor signaling pathway |
| *stat3* | Signal transducer and activator of transcription 3 (acute-phase response factor) | 0.633 | 2.44E-03 | Necroptosis |
| *h2ax1* | H2A.X variant histone family member 1 | 0.806 | 2.52E-03 | Necroptosis |

| *jak2a* | Janus kinase 2a | 0.589 | 3.45E-03 | Necroptosis; Herpes simplex virus 1 infection |
| --- | --- | --- | --- | --- |
| *stat6* | Signal transducer and activator of transcription 6, interleukin-4 induced | 0.660 | 6.05E-03 | Necroptosis |
| *faslg* | Fas ligand (TNF superfamily, member 6) | 2.985 | 3.36E-02 | Necroptosis; Herpes simplex virus 1 infection |
| *stat4* | Signal transducer and activator of transcription 4 | 0.810 | 3.60E-02 | Necroptosis; Apoptosis |
| *parp1* | Poly (ADP-ribose) polymerase 1 | -0.684 | 8.84E-03 | Necroptosis; Base excision repair; Apoptosis |
| *tradd* | Tnfrsf1a-associated via death domain | -0.623 | 1.11E-02 | Necroptosis; Herpes simplex virus 1 infection; RIG-I-like receptor signaling pathway; Apoptosis |
| *capn1b* | Calpain 1, (mu/I) large subunit b | -1.626 | 1.84E-02 | Necroptosis; Apoptosis |
| *ppid* | Peptidylprolyl isomerase D | -0.635 | 3.33E-02 | Necroptosis |
| *irf3* | Interferon regulatory factor 3 | 1.187 | 3.23E-03 | Herpes simplex virus 1 infection; RIG-I-like receptor signaling pathway |
| *irf7* | Interferon regulatory factor 7 | 0.928 | 2.81E-03 | Herpes simplex virus 1 infection; RIG-I-like receptor signaling pathway; NOD-like receptor signaling pathway |
| *cgasa* | Cyclic GMP-AMP synthase a | 0.854 | 6.41E-03 | Herpes simplex virus 1 infection |
| *tap2a* | Transporter associated with antigen processing, subunit type a | 1.017 | 1.37E-02 | Herpes simplex virus 1 infection |
| *tapbp.1* | TAP binding protein (tapasin), tandem duplicate 2 | 0.739 | 1.50E-02 | Herpes simplex virus 1 infection |
| *socs3b* | Suppressor of cytokine signaling 3b | 0.677 | 2.04E-02 | Herpes simplex virus 1 infection |
| *ifih1* | Interferon induced with helicase C domain 1 | 0.701 | 3.63E-02 | Herpes simplex virus 1 infection |
| *rad54l* | RAD54 like | -2.140 | 9.02E-04 | Homologous recombination |
| *eme1* | Essential meiotic structure-specific endonuclease 1 | -1.827 | 9.69E-04 | Homologous recombination; Fanconi anemia pathway |
| *bard1* | BRCA1 associated RING domain 1 | -1.504 | 2.23E-02 | Homologous recombination |
| *parp2* | Poly (ADP-ribose) polymerase 2 | -0.947 | 4.57E-02 | Base excision repair; Apoptosis |
| *mus81* | MUS81 structure-specific endonuclease subunit | -0.962 | 2.73E-02 | Homologous recombination; Fanconi anemia pathway |
| *dhx58* | DEXH (Asp-Glu-X-His) box polypeptide 58 | 2.455 | 2.39E-03 | RIG-I-like receptor signaling pathway |
| *trim25* | Tripartite motif containing 25 | 1.069 | 2.51E-03 | RIG-I-like receptor signaling pathway |
| *tank* | RAF family member-associated NFKB activator [ | 0.873 | 1.00E-02 | RIG-I-like receptor signaling pathway; NOD-like receptor signaling pathway |
| *fanca* | FA complementation group A | -0.663 | 3.28E-02 | Fanconi anemia pathway |
| *fancd2* | FA complementation group D2 | -1.077 | 4.79E-02 | Fanconi anemia pathway |
| *uckl1b* | Uridine-cytidine kinase 1-like 1b | 0.979 | 1.06E-02 | Drug metabolism - other enzymes |
| *Gstr* | Glutathione S-transferase rho | -0.471 | 1.15E-02 | Drug metabolism - other enzymes |
| *gusb* | Glucuronidase, beta | -0.405 | 2.06E-02 | Drug metabolism - other enzymes; Pentose and glucuronate interconversions |
| *ugt5d1* | UDP glucuronosyltransferase 5 family, polypeptide D1 | -0.566 | 4.82E-02 | Drug metabolism - other enzymes; Pentose and glucuronate interconversions |
| *dcxr* | Dicarbonyl/L-xylulose reductase | -0.580 | 3.80E-03 | Pentose and glucuronate interconversions |
| *Kl* | Klotho | -0.533 | 4.09E-02 | Pentose and glucuronate interconversions |
| *Xylb* | Xylulokinase homolog (H. influenzae) | -0.381 | 4.69E-02 | Pentose and glucuronate interconversions |
| *ca6* | Carbonic anhydrase VI | 1.272 | 2.44E-03 | Nitrogen metabolism |
| *cahz* | Carbonic anhydrase | 1.071 | 5.41E-03 | Nitrogen metabolism |
| *ca4c* | Carbonic anhydrase IV c | -4.501 | 1.09E-02 | Nitrogen metabolism |
| *tp53bp1* | Tumor protein p53 binding protein, 1 | -0.962 | 3.98E-02 | NOD-like receptor signaling pathway |
| *nat10* | N-acetyltransferase 10 | -0.949 | 1.32E-02 | Ribosome biogenesis in eukaryotes |
| *xpo1a* | Exportin 1 (CRM1 homolog, yeast) a | -0.674 | 1.65E-02 | Ribosome biogenesis in eukaryotes |
| *wdr3* | WD repeat domain 3 | -0.743 | 2.56E-02 | Ribosome biogenesis in eukaryotes |
| *rrp7a* | Ribosomal RNA processing 7 | -0.708 | 4.07E-02 | Ribosome biogenesis in eukaryotes |
| *fosab* | V-fos FBJ murine osteosarcoma viral oncogene homolog Ab | 1.822 | 2.09E-04 | Apoptosis |
| *map3k14a* | Mitogen-activated protein kinase kinase kinase 14a | 0.583 | 1.20E-02 | Apoptosis |
| *ddit3* | DNA-damage-inducible transcript 3 | 0.546 | 4.94E-02 | Apoptosis |

**Supplementary Table S5** Differentially expressed genes (DEGs) between FT2RF6 and CON in significantly enriched KEGG pathways (*p-value* < 0.05)

| Gene | Description | Log_2_FC | *P*-value | KEGG Pathway |
| --- | --- | --- | --- | --- |
| *cahz* | Carbonic anhydrase | 0.701 | 4.85E-02 | Nitrogen metabolism |
| *ca4c* | Carbonic anhydrase IV c | -3.610 | 2.58E-02 | Nitrogen metabolism |
| *ca15b* | Carbonic anhydrase XV c | -1.772 | 4.13E-02 | Nitrogen metabolism |
| *dffb* | DNA fragmentation factor, beta polypeptide (caspase-activated DNase) | 1.013 | 1.86E-02 | Apoptosis |
| *map3k14a* | Mitogen-activated protein kinase kinase kinase 14a | 0.527 | 3.95E-02 | Apoptosis |
| *gadd45ga* | Growth arrest and DNA-damage-inducible, gamma a | 0.945 | 4.89E-02 | Apoptosis |
| *capn1b* | Calpain 1, (mu/I) large subunit b | -3.947 | 5.46E-05 | Apoptosis |
| *Atm* | ATM serine/threonine kinase | -0.728 | 4.12E-02 | Apoptosis |
| *pik3r1* | Phosphoinositide-3-kinase regulatory subunit 1 | -0.411 | 4.13E-02 | Apoptosis |
| *pidd1* | P53-induced death domain protein 1 | -1.107 | 4.93E-02 | Apoptosis |
| *acss1* | Acyl-CoA synthetase short chain family member 1 | 0.640 | 2.44E-02 | Glyoxylate and dicarboxylate metabolism; Pyruvate metabolism |
| *wtap* | WT1 associated protein | -1.068 | 2.30E-02 | Glyoxylate and dicarboxylate metabolism; Pyruvate metabolism |
| *mdh1* | Malate dehydrogenase 1 | -2.214 | 4.81E-02 | Glyoxylate and dicarboxylate metabolism; Pyruvate metabolism |
| *mcm6* | Minichromosome maintenance complex component 6 | -0.985 | 1.44E-02 | DNA replication |
| *mcm5* | Minichromosome maintenance complex component 5 | -1.134 | 2.48E-02 | DNA replication |
| *pole* | Polymerase (DNA directed), epsilon | -0.990 | 3.89E-02 | DNA replication |
| *pals1b* | Protein associated with LIN7 1, MAGUK p55 family member b | 1.272 | 3.27E-02 | Tight junction |
| *cldn11a* | Claudin 11a | -1.998 | 3.54E-02 | Tight junction |
| *scd* | Stearoyl-CoA desaturase | -1.524 | 2.43E-03 | PPAR signaling pathway |
| *acsl3a* | Acyl-CoA synthetase long chain family member 3a | -1.570 | 9.54E-03 | PPAR signaling pathway |

**Supplementary Table S6** Differentially expressed genes (DEGs) between FT3RF9 and CON in significantly enriched KEGG pathways (*p-value* < 0.05).

| Gene | Description | Log_2_FC | *P*-value | KEGG Pathway |
| --- | --- | --- | --- | --- |
| *fads2* | Fatty acid desaturase 2 | 3.334 | 3.25E-03 | PPAR signaling pathway |
| *acsl4a* | Acyl-CoA synthetase long chain family member 4a | 0.859 | 1.69E-02 | PPAR signaling pathway |
| *angptl4* | Angiopoietin-like 4 | -1.020 | 1.54E-03 | PPAR signaling pathway |
| *lpla* | Lipoprotein lipase a | -0.632 | 4.13E-02 | PPAR signaling pathway |
| *cd36* | CD36 molecule (CD36 blood group) | -1.391 | 3.25E-02 | PPAR signaling pathway |
| *nop56* | NOP56 ribonucleoprotein homolog | -0.687 | 4.41E-03 | Ribosome biogenesis in eukaryotes |
| *gnl2* | G protein nucleolar 2 | -0.492 | 1.35E-02 | Ribosome biogenesis in eukaryotes |
| *afg2a* | AFG2 AAA ATPase homolog A | -0.429 | 2.57E-02 | Ribosome biogenesis in eukaryotes |
| *utp4* | UTP4 small subunit processome component | -0.566 | 3.49E-02 | Ribosome biogenesis in eukaryotes |
| *rrp7a* | Ribosomal RNA processing 7 homolog A | -0.631 | 4.92E-02 | Ribosome biogenesis in eukaryotes |
| *cd99* | CD99 molecule | 0.602 | 2.15E-03 | Cell adhesion molecules |
| *cldn15la* | Claudin 15-like a | -4.053 | 3.05E-03 | Cell adhesion molecules |
| *cldn33b* | Claudin 33b | -3.734 | 1.75E-02 | Cell adhesion molecules |
| *cntn1b* | Contactin 1b | -2.745 | 1.89E-02 | Cell adhesion molecules |
| *cldn11a* | Claudin 11a | -2.099 | 2.59E-02 | Cell adhesion molecules |
| *cadm3* | Cell adhesion molecule 3 | -3.459 | 3.76E-02 | Cell adhesion molecules |
| *prlra* | Prolactin receptor a | 0.866 | 4.08E-02 | Cytokine-cytokine receptor interaction |
| *cxcl19* | Chemokine (C-X-C motif) ligand 19 | 1.085 | 4.54E-02 | Cytokine-cytokine receptor interaction |
| *epor* | Erythropoietin receptor | 0.850 | 4.86E-02 | Cytokine-cytokine receptor interaction |
| *tnfrsf11a* | Tumor necrosis factor receptor superfamily, member 11a, NFKB activator | -5.222 | 4.10E-03 | Cytokine-cytokine receptor interaction |
| *tgfbr1b* | Transforming growth factor, beta receptor 1 b | -0.507 | 1.93E-02 | Cytokine-cytokine receptor interaction |
| *bmp3* | Bone morphogenetic protein 3 | -1.554 | 2.42E-02 | Cytokine-cytokine receptor interaction |
| *tnfrsf21* | Tumor necrosis factor receptor superfamily, member 21 | -2.112 | 2.77E-02 | Cytokine-cytokine receptor interaction |
| *ebi3* | Epstein-Barr virus induced 3 | -1.002 | 3.67E-02 | Cytokine-cytokine receptor interaction |
| *tnfsf13b* | TNF superfamily member 13b | -1.009 | 4.85E-02 | Cytokine-cytokine receptor interaction |
| *amacr* | Alpha-methylacyl-CoA racemase | -0.527 | 2.70E-03 | Primary bile acid biosynthesis |
| *sdsl* | Serine dehydratase-like | 0.726 | 3.16E-02 | Glycine, serine and threonine metabolism |
| *agxtb* | Alanine--glyoxylate and serine--pyruvate aminotransferase b | -0.694 | 2.56E-02 | Glycine, serine and threonine metabolism |
| *pigh* | Phosphatidylinositol glycan anchor biosynthesis, class H | 1.317 | 2.33E-03 | Glycosylphosphatidylinositol (GPI)-anchor biosynthesis |
| *pigv* | Phosphatidylinositol glycan anchor biosynthesis, class V | -0.995 | 2.14E-02 | Glycosylphosphatidylinositol (GPI)-anchor biosynthesis |
| *papss2a* | 3, -phosphoadenosine 5, -phosphosulfate synthase 2a | -0.541 | 1.86E-02 | Sulfur metabolism |
